# Andrographolide Selectively Inhibits the Growth of LA7 Mammary Adenocarcinoma Cells

**DOI:** 10.1021/acsomega.5c04617

**Published:** 2025-11-20

**Authors:** Kallol Roy, Parthiv Kar, Saikat Haldar, Sarangthem Dinamani Singh, Selvaraman Nagamani, Himangsu K Bora, Binoy K Saikia, Rituraj Konwar

**Affiliations:** 1 Centre for Preclinical Studies (CPS), Biological Sciences and Technology Division (BSTD), 81721 CSIR-North East Institute of Science and Technology (CSIR-NEIST), Jorhat 785006, Assam, India; 2 Advanced Computation and Data Sciences Division (ACDSD), 81721 CSIR-North East Institute of Science and Technology (CSIR-NEIST), Jorhat 785006, Assam, India; 3 Coal and Energy Group (C&E), 81721 CSIR-North East Institute of Science and Technology (CSIR-NEIST), Jorhat 785006, Assam, India; 4 AcSIR-Academy of Scientific and Innovative Research, Ghaziabad, Uttar Pradesh 201002, India

## Abstract

Breast cancer (BC) causes significant mortality and morbidity, specifically in women globally. The first line of available therapies are far from satisfactory due to their adverse side effects, drug resistance, and reoccurrence of the cancer. As a result, natural source-based complementary medicines with anticancer activity and lesser side effects are widely investigated. Andrographolide (AGL), an active phytomolecule of *Andrographis paniculata*, exhibits diverse pharmacological activities against various diseases. The purpose of the study is to investigate the potential anticancer activity of AGL on LA7 cells, an *in vitro* mammary tumor model system, to understand its interaction with red blood cells (RBC) and effect on their membrane integrity. LA7 is a mammary adenocarcinoma cell line established by Renato Dulbecco in 1979 from the mammary tumor of Sprague–Dawley (SD) rat treated with 7,12-dimethylbenz­[*a*]­anthracene (DMBA). At first, a simple, rapid solvent extraction-gravimetric column chromatography method was employed for separation and purification of AGL from the dried leaf extract of *A. paniculata*. AGL showed significant anticancer activities on LA7 cells, with a potency similar to tamoxifen, an FDA-approved drug for BC. Mechanistic investigation showed that AGL triggers DNA damage, disruption of mitochondrial integrity, oxidative stress, cell cycle arrest at the G2/M phase, and apoptosis in LA7 mammary tumor cells. Furthermore, computational studies were performed to study the atomistic level understanding of AGL targeting LA7 cells. AGL exhibited a strong stable interaction with the active sites of BCL-2, NF-κB, and PKC-α proteins. Hematological complications are common side effects of most of the chemotherapeutic drugs. Toxicity analysis using Sprague–Dawley (SD) rats and human blood cells demonstrated no deleterious effects of AGL and devoid of hemolysis of RBCs. Morphological analysis by light and scanning electron microscopy (SEM) showed no observable changes in structural integrity of RBC membranes indicating nonhemolytic nature of AGL. Our results suggested that AGL has significant potential to be explored as an anticancer agent in BC therapy without any RBC toxicity.

## Graphical abstract

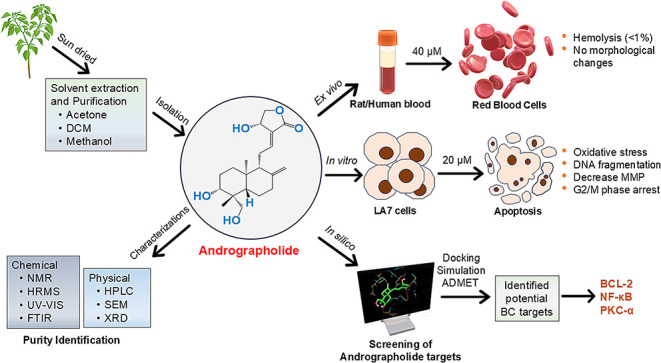

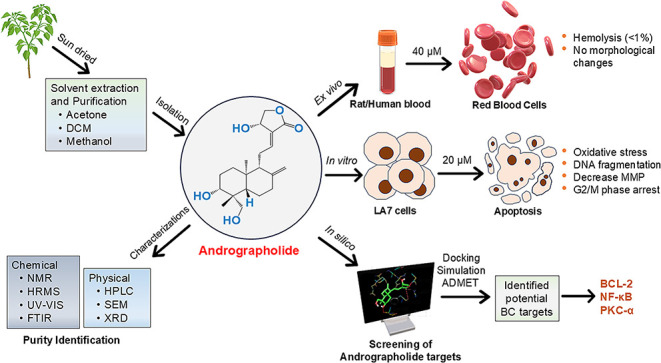

## 1 Introduction

Breast cancer (BC) is the most frequently diagnosed life-threatening disease in women globally, accounting for 32% of cases among all cancers in 2024. BC is often resistant to the first line of chemotherapy, endocrine therapy, and targeted therapy, and undesirable side effects cause major obstacles in BC treatment.^1,2^ LA7 is a mammary adenocarcinoma cell line established in 1979 by Renato Dulbecco from mammary tumors of Sprague–Dawley (SD) rats treated with 7,12-dimethylbenz­[*a*]­anthracene (DMBA). The application of LA7 in mammary tumor model development is widely used with a short latency period, which is advantageous for quicker assessment of novel compounds for the advanced stage of carcinogenesis through chemoprevention, endocrine therapy, and other therapies against BC.^3^ Moreover, LA7 cells exhibit several similarities to human BC particularly in morphological features and intracellular components and can generate heterogeneous luminal epithelial, myoepithelial, and alveolar cell lineage that recapitulates morphological and functional properties of the ductal-alveolar framework of the mammary gland for evaluating potential BC therapies.^4,5^ Few earlier studies have demonstrated the suppressive activity of synthetic compounds or drugs against LA7-induced tumors, although side effects and resistance remain a common challenge.^2,6–8^ Further different plant-based leaf extracts and β-mangostin, a xanthone compound, were also reported to produce antiproliferative effects in LA7 cell proliferation.^3,9,10^ As a result, there is an increasing interest in the study of compounds derived from natural sources with anticancer activity in BC.

Andrographolide (AGL), a major bioactive phytomolecule of*Andrographis paniculata* (Burm.f.) Nees plant with molecular formula C_20_H_30_O_5_ (Figure 1A), exhibits diverse pharmacological properties including anti-inflammatory, antihyperglycemic, hepatoprotective, antimicrobial, antifertility, and anticancer activity.^11–13^ In the past, different extraction methods for AGL from *A. paniculata* were reported, which were tedious, were time-consuming, and retained significant impurities.^14–16^ Therefore, the design of a cost-effective, efficient extraction technique has always remained important before any pharmaceutical or other applications. Earlier studies reported the anticancer activity of AGL against different types of human BC such as T-47D, BT549, MCF-7, and MDA-MB-231 cells by inhibition of cell cycle progression, affecting angiogenesis, generation of oxidative injury, or down-regulation of stemness-related proteins.^17–19^ Recent investigations demonstrated that AGL triggers ROS-FOXM1, ER-α, and PI3K/AKT/mTOR signaling pathways to suppress cancer cell growth and malignancy.^20,21^ However, no study has been conducted so far for the effects of AGL on the SD rat-originated LA7 mammary tumor cell line. Though LA7 exhibits similarity with human breast cancer cells, it remains unknown if there could be species-specific differences. At the same time, it is also important to know the possible side effects of therapeutic leads on the cell membrane, which directly reflects safety or toxicity of the lead compound.^22^ The red blood cell (RBC) is considered as an ideal model system for a basic understanding of the interaction between drug and cell membrane. RBCs have a simplified structure compared to other cell types, because of the lack of any internal organelle, and play a crucial role in gas-ion transport to the cell membrane. Membrane deformity and hemoglobin content release from RBC is well correlated with toxicity of a drug or a lead compound.^23,24^ So far, only one report demonstrated lack of hemolytic activity of AGL in RBCs measured by UV–vis spectroscopy.^25^ However, the preliminary study did not report the status of specific morphological and membrane effects of AGL–RBC interaction at the microscopic level. The cell membrane of RBC is very unique and delicate, which helps RBCs to travel through narrow capillaries and is very sensitive to thermal and chemical assaults. Lysis of the cell membrane of RBC is the critical stage of pathological conditions involving RBCs, and often alterations of RBC morphology strongly correlate with several hemopathies; therefore, it is important to understand the effect of the candidate compound on RBC morphology as an indicator of its detrimental effects. Hence, an in-depth study between AGL–RBC membrane interactions is necessary to elucidate any possible side effects of AGL treatment on BC.

**Figure 1:**
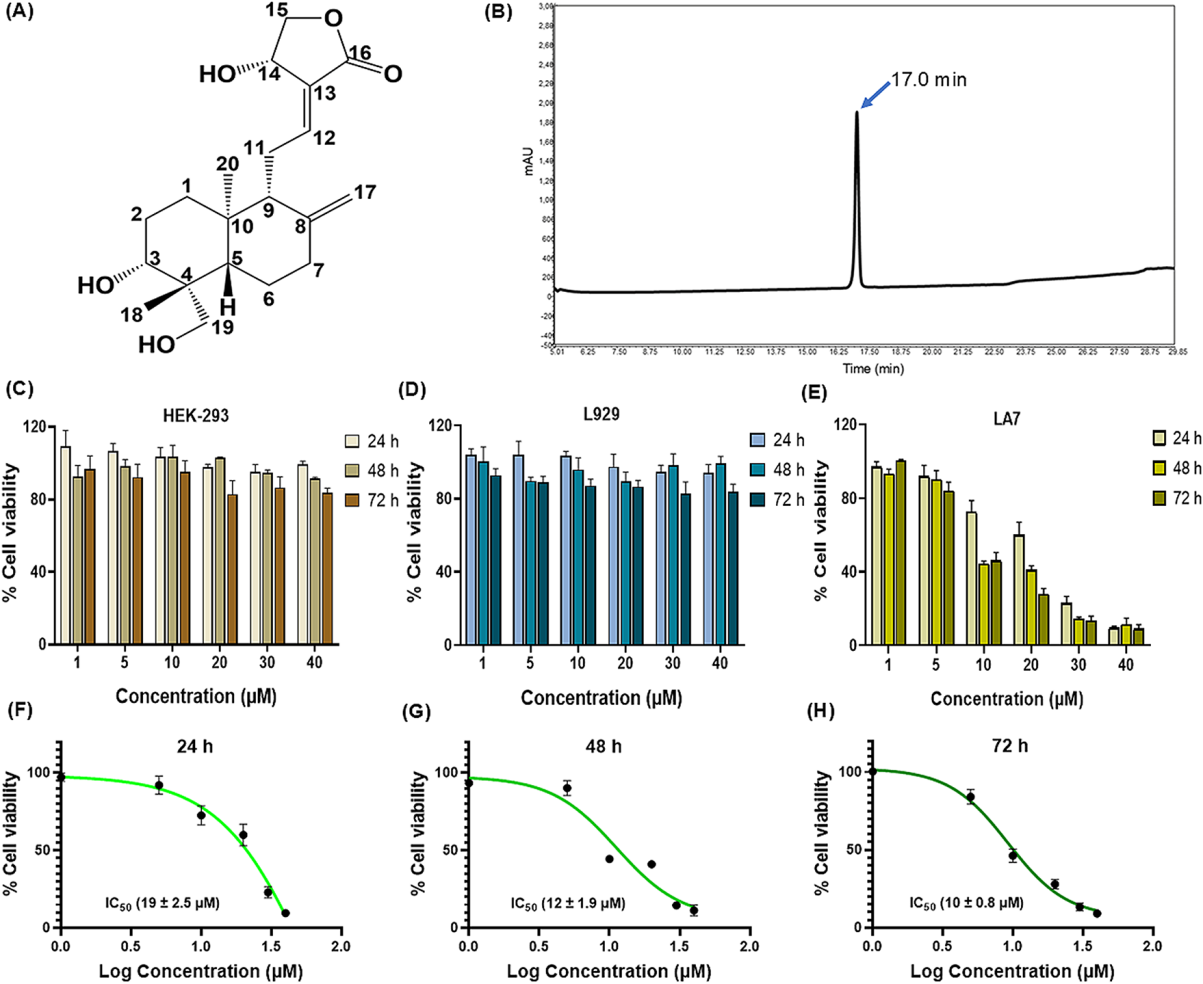
(A) 2D chemical structure of AGL. (B) HPLC chromatogram of AGL isolated from *Andrographis paniculata* showed a single sharp peak at *R*_t_ 17.0 min. Dose- and time-dependent effects of AGL on (C) HEK-293, (D) L929 normal, and (E) LA7 mammary tumor cells by the MTT assay method. The percent (%) cell viability was calculated with respect to control. Dose-responsive curve to determine the IC_50_ values of AGL on LA7 cells at (F) 24 h, (G) 48 h, and (H) 72 h. Data presented as mean of three independent experiments with SD (*n* = 9).

In this study, a simple solvent extraction gravimetric column chromatography method was applied to isolate and purify AGL from crude leaf extract of *A. paniculata* and examined the anticancer activity in LA7 cells analyzed through *in vitro* and *in silico* studies. In order to assess hematological toxicity, we investigated the effects of AGL on the membrane integrity as well as possible alteration of morphology of RBCs.

## 2 Materials and Methods

### 2.1 Chemicals

All the solvents (acetone, dichloromethane, and methanol) were procured from Merck Co. (Darmstadt, Germany). 3-(4,5-Dimethylthiazol-2-yl)-2,5-diphenyltetrazolium bromide (MTT), 4′,6-diamidino-2-phenylindole (DAPI), and acridine orange (AO) were purchased from HiMedia Laboratories Private Limited (India). Dulbecco’s modified Eagle’s medium (DMEM), fetal bovine serum (FBS), antibiotic–antimycotic solution, 2′,7′-dichlorodihydrofluorescein diacetate (DCFH-DA), tamoxifen, and propidium iodide (PI) were procured from Sigma-Aldrich (India). 5,5,6,6′-Tetrachloro-1,1′,3,3′-tetraethylbenzimidazoylcarbocyanine iodide (JC-1) was obtained from Thermo Fisher Scientific (India).

### 2.2 Taxonomic Identification, Collection, and Processing of *A. paniculata*

The plant sample was authenticated on the basis of its morphological features. Flowering herb: an erect or procumbent annual, profusely branched, herbaceous plant, height around 30–50 cm.^26,27^ Habitat: moist soil, shady, and grows in isolated patches. Leaf adaxial surface: simple, dark green, lanceolate, exstipulate, opposite, long 2–12 cm, wide 1–3 cm, bractiform with glabrous short petiole. Leaf abaxial surface: pale green. Leaf shape: ovate with attenuated base and obtuse apex. Leaf venation: pinnate craspedodromous.^26,28^ Inflorescence: rachis, 10–30 mm long, axillary, and terminal panicle. Bracts: small, acicular, 2.5 mm long. Pedicel: short, slender.^26^ Calyx: 5, small, linear; calyx lobes subacute 3 mm long, glandular. Corolla: 6 mm long, bilabiate; white with yellowish gradient on oblong upper lip, broadly cuneate lower lip splashed with a violet streak, lobes imbricated in the bud. Stamens: 2 in number, inserted in the throat of the corolla tube, basally bearded, exerted, or included. Filaments: ciliated. Ovary: superior, two-celled; style minutely exserted. Fruits: capsule, linear to oblong, gradually acute at both ends, having numerous small, subquadrate, pitted seeds.^26,27,29^ Stem: deep green, solitary, upper part quadrangular, lower part nearly rounded with longitudinal furrows. Root: fleshy, fusiform, taproot system.

Mature dark-green leaves of *A. paniculata* (Burm.f.) Nees were collected in June 2023 from Mainaguri, Jalpaiguri, West Bengal, India. The voucher specimen of *A. paniculata* was deposited at the herbarium of CSIR-North East Institute of Science and Technology, Jorhat, India (voucher specimen no. 0834). The leaves were thoroughly washed under running water and kept for 2 days for sun-drying. The dried leaves were coarsely ground by using a thoroughly cleaned kitchen grinder.

### 2.3 Preparation of the Extract

Dried powder of the leaves (150 g) was subjected to solvent extraction using acetone (2 × 750 mL) under stirring conditions for 24 h. The organic layers were decanted, pooled, and subjected to filtration using Whatman filter paper (Grade 1). Furthermore, acetone was removed in a rotary evaporator (42 °C, 250 mbar) to obtain 5.30 g (3.5% w/w) dark-green semi-solid extract.

### 2.4 Purification and Characterizations of AGL

The extract was subjected to fractionation and purification through repeated gravity column chromatography. The extract (4.89 g) was loaded on a silica gel column (60–120 mesh, L × D of 22 cm × 3.5 cm) and eluted with 3.5 L of dichloromethane followed by 4.5 L of 2% methanol in dichloromethane. An AGL-rich fraction was eluted with 2% methanol in dichloromethane as monitored through thin layer chromatography (TLC). The fractions containing AGL were pooled and concentrated to obtain 1.14 g of deep-green solid. It was further transferred to another silica gel column (60–120 mesh, L × D of 18 cm × 2.0 cm) eluting with 1.9 L of 2% methanol in dichloromethane. AGL-rich fractions were similarly pooled and concentrated to obtain 0.96 g of greenish-white solid. It was washed with excess dichloromethane to eliminate residual pigments and minor impurities to furnish 0.78 g of pure AGL as off-white powder.

The purified AGL was characterized by proton (^1^H) and carbon (^13^C) nuclear magnetic resonance (NMR) spectroscopy and high-resolution mass spectrometry (HRMS) data. ^1^H NMR data were recorded at 400 MHz and^13^C NMR data recorded at 100 MHz in an FT-NMR spectrometer (JEOL, USA). The sample was prepared in deuterated DMSO, and residual solvent peaks at 2.5 and 39.52 ppm were used as the reference of ^1^H and ^13^C NMR analyses, respectively. The obtained results were processed in MestReNova software. Molecular mass of isolated AGL was measured by a mass spectrometer (Xevo G2-XS QTof, Water, Austria) using ESI as the ionization source. Purity of the isolated metabolite was also confirmed through HPLC analysis (Dionex UltiMate 3000). Furthermore, the absorption spectra of isolated AGL were determined by a UV–vis spectrophotometer (Labindia 1000+). FTIR analysis was performed to determine surface functional groups using a Fourier transform infrared spectrophotometer (Spectrum 2, PerkinElmer). Crystallinity of AGL was studied via an X-ray powder diffractometer (Rigaku Ultima IV) with a Cu Kα line as a source of radiation (λ = 0.15418 nm) and scanned over a 2θ range of 3–80° with a step angle of 0.02°. SEM images were recorded using a field-emission scanning electron microscope (SIGMA, Carl ZEISS Microscopy, Germany).

### 2.5 Cell Culture

Normal cell lines, human embryonic kidney-293 (HEK-293) and NCTC clone 929 (L929) stocks, were available in the laboratory, and rat mammary tumor cell line LA7 was obtained from ATCC, USA (CRL-2283). All the cells were maintained in high-glucose DMEM supplemented with 10% FBS, 100 U/mL penicillin, and 100 μg/mL streptomycin at 37 °C temperature in a humidified environment with 5% (v/v) CO_2_.

### 2.6 Cellular Viability Assay

The viability of cells with AGL treatment was measured by MTT assay, as described earlier.^30^ Initially, 5 × 10^3^ cells were seeded into each 96-well plate overnight to reach 80% confluency. Cells were treated with AGL of varying concentrations (1–40 μM) for 24, 48, and 72 h. After that, 10 μL of MTT (12 mM) was added in fresh 100 μL of media and incubated for 2 h 30 min under dark conditions. Finally, formazan crystals were solubilized in 100 μL of DMSO by keeping 20 min in a plate shaker and absorbance was evaluated at 540 nm using a microplate reader (Biotek, Cytation 5). The IC_50_ value of the AGL at three-time durations were determined using GraphPad Prism Version 8.

### 2.7 Clonogenic Assay

The colony formation potential of LA7 mammary tumor cells was assessed by *in vitro* anchorage-dependent clonogenic assay.^31^ Briefly, 2 × 10^3^ cells were seeded into each 60 mm culture dish and allowed to adhere overnight. Cells were treated with 10, 20, and 30 μM concentrations of AGL for 24 h. Thereafter, cells were washed and replenished with fresh complete medium changing every 3 days and allowing them to grow for the next 10 days. After the end of incubation, LA7 cell colonies were subjected to 4% paraformaldehyde fixation and staining for 30 min with 0.5% crystal violet stain. After being stained, culture dishes were rinsed in tap water and dried at room temperature. Finally, images were recorded to determine the effect of AGL on the single-cell colony forming efficiency of LA7 cells.

### 2.8 Cell Migration Assay

The migration behavior of LA7 cells after AGL treatment was analyzed by cell monolayer scratch assay with some minor modifications.^32^ For this, 5 × 10^5^ cells were seeded in 60 mm cell culture dishes for 24 h to attain 80% cell monolayer confluency. A scratch was made with a sterile 200 μL pipette tip in the cell monolayer in the center of each dish and treated with or without AGL (10 and 20 μM) for 24 h. Images of scratched area of the cell monolayer were recorded and analyzed for cell migration status in the scratched area using ImageJ software.

### 2.9 Staining of Nuclei of LA7 Cells

Nuclear dye DAPI has strong binding affinity to the A–T-rich sequence of DNA and emits fluorescence upon binding to DNA. Therefore, it is routinely used for visualizing the nuclear morphology of cells or as a counter stain.^33^ Briefly, 10 and 20 μM concentrations of AGL were administered to a confluent LA7 cell monolayer grown in a 96-well cell culture plate and incubated for 24 h. Subsequently, cells were rinsed with PBS followed by 15 min of 4% paraformaldehyde fixation under low light conditions. Following that, DAPI was added to stain cells for 30 min under dark conditions, and images of LA7 cell nuclei were recorded using fluorescence microscopy.

### 2.10 Mitochondrial Membrane Integrity

JC-1 dye is widely used for determining mitochondrial integrity via alteration of mitochondrial membrane potential (MMP).^34^ LA7 cells were subjected to treatment of 10 and 20 μM concentrations of AGL for 24 h. After AGL treatment, LA7 cells were incubated with a 5 μg/mL concentration of DAPI in PBS under dark conditions for 30 min, followed by treatment of LA7 cells with a 10 μg/mL concentration of JC-1 dye and further incubated for 30 min. Finally, cells were rinsed in PBS and each treatment group’s images were recorded under a fluorescence microscope (Axio Vert. A1).

### 2.11 Assessment of Reactive Oxygen Species (ROS) Generation

The intracellular ROS level in the LA7 cell line following treatment with AGL was determined using DCFH-DA dye.^30^ For this, 2 × 10^5^ cells were seeded in a six-well plate and allowed to grow at 37 °C in a CO_2_ incubator. The next day, 10 and 20 μM concentrations of AGL were added to the culture media and cells were incubated for a further 24 h. The highest concentration of AGL (20μM) was treated along with 100 μM of *N*-acetyl cysteine (NAC), a ROS scavenger and 100 μM of H_2_O_2_ , a ROS inducer was used as a positive control. After treatment, cells were further incubated for 30 min in PBS medium containing 500 μL of DCFH-DA (10 μM) and recorded with a fluorescence microscope (Axio Vert. A1).

For the determination of dose-dependent change in ROS level following incubation with DCFH-DA dye, LA7 cells were incubated in 300 μL of chilled radioimmunoprecipitation assay (RIPA) buffer for 15 min. Then, lysed cells were centrifuged at 21,130*g* for 10 min at 4 °C followed by transferring of the supernatant into a black-bottom 96-well plate, and DCFH-DA intensity (excitation: 485 nm, emission: 530 nm) was recorded with a microplate reader (Biotek, Cytation 5).

### 2.12 Acridine Orange (AO) and Propidium Iodide (PI) Staining

The AO-PI dual fluorescent staining is routinely used for visualization of cellular and nuclear changes occurring during apoptosis.^35^ For this, LA7 cells were seeded into 96-well plates and allowed to grow for 24 h at 37 °C in a CO_2_ incubator. Cells were incubated with 10 and 20 μM concentrations of AGL along with a 20 μM concentration of tamoxifen (TAM) for another 24 h. 100 μL of 4% paraformaldehyde was used for fixing the cells followed by incubation with a mixture of AO (100 μg/mL) and PI (50 μg/mL) in PBS for 10 min. The cellular and nuclear changes were visualized and recorded under a fluorescence microscope (Axio Vert. A1).

### 2.13 Cell Cycle Analysis

LA7 cells were incubated with 5 and 10 μM concentrations of AGL for 24 h, and its effects on cell cycle progression were determined by flow cytometric analysis using PI staining.^36^ Following treatment, cells were harvested and fixed with chilled 70% ethanol and stored overnight at 4 °C. Then, cell pellets were resuspended in 100 μg/mL of RNase A for 1 h followed by incubation with 100 μg/mL of PI in PBS for 30 min at room temperature. Then, different cell cycle phases of the stained cells were deermined and recorded with a flow cytometer (CytoFLEX S, Beckman Coulter).

### 2.14 Computational Studies

Additionally, we used different computational methods to identify the possible drug targets of AGL using structure- and ligand-based approaches.

#### 2.14.1 Screening of Possible Targets of AGL

Three different servers, namely, SwissTargetPrediction (http://www.swisstargetprediction.ch/), Biological Activity Spectrum server (PASS analysis) (https://www.way2drug.com/passonline/), and DIGEP-Pred 2.0 (https://www.way2drug.com/digep-pred/) web server, were used to identify the possible targets of AGL. The SMILES string of the AGL was obtained from the PubChem database (https://pubchem.ncbi.nlm.nih.gov/compound/5318517) and given as an input in these three servers. These servers apply ligand similarity and quantitative structure activity relationship (QSAR) approaches to identify the possible targets for the given input (i.e., AGL). Additionally, we have collected LA7-specific expressed genes from existing literature (Table S1). We considered the proteins that are identified commonly among these three methods as the possible targets for AGL.

#### 2.14.2 Molecular Docking

The AGL was docked in the active site of three selected proteins, namely, BCL-2 (PDB ID: 4LVT), NF-κB (PDB ID: 1NFI), and PKC-α (PDB ID: 3IW4). The structures of these proteins were downloaded from the Protein Data Bank (https://www.rcsb.org/). Hydrogens at pH 7.4 and Gasterier charges were added, and the protein geometry was optimized and energy minimized using AutoDock Tools 1.5.7. The molecular docking was performed in AutoDock, and the results were analyzed in PyMOL.

#### 2.14.3 Molecular Dynamics (MD) Simulation

The best docking conformer from each protein was subjected to MD simulation using Gromacs V 2021.4 with a CHARMM force field and a TIP3P water molecule. The topological parameters of the AGL were generated using the CGenFF server. MD simulations were performed under periodic boundary conditions (PBCs) with a cubical box maintaining distances of 1.0 and 1.5 nm and the boundary edges between the protein and drug complexes depending on the size of the proteins. The protein–drug complexes were solvated with SPC water molecules, and the system was neutralized by adding the counterions into the solvated box depending on the charge of the system. Each system was equilibrated by running 100 ps of the NVT and NPT ensembles. 0.15 M NaCl was added to the system. A total of 100 ns MD simulation was performed on each system, and trajectories were recorded at every 0.002 ps. The MD trajectories were further performed to analyze the root means square deviation (RMSD) of protein backbone/ligand and total number of hydrogen bonds. The binding free energies of the AGL in three different proteins were evaluated using the gmx_MMPBSA tool.^37,38^ The last 20 ns MD trajectories from the total production MD (100 ns) was extracted and analyzed for the binding energy assessment.

#### 2.14.4 ADMET Analysis

The absorption, distribution, metabolism, excretion, and toxicity (ADMET) profiling of AGL was estimated using the online tool ADMETlab 3.0.

### 2.15 Blood Collection and RBC Isolation

The blood samples were collected with prior ethical approval from the Institutional Animal Ethics Committee (IAEC) and the Institutional Human Ethics Committee (IHEC) at CSIR-NEIST, Jorhat, Assam. The blood samples were collected from three Sprague–Dawley (SD) rats (approval ref no. CSIR/NEIST/IAEC/02/23/007) from the median canthus region of the eye, while human blood samples were collected with prior ethical and informed consent from healthy volunteers (approval ref no. IHEC/NEIST/14 June 2024/04/06/008/RK) by venipuncture in K3-EDTA-containing tubes. Whole blood was subjected to centrifugation with 410*g* at 4 °C for 10 min, to isolate RBCs that were further washed thrice in isotonic PBS solution to remove the remaining traces of plasma.^39^

### 2.16 Hemolysis Assay

The cleaned RBC pellet was resuspended in PBS to make 4% RBC suspension for the hemolysis test.^39^ In the microcentrifuge tube, 0.5 mL of 4% RBCs suspension was added with the equal volume of AGL to make desired final concentrations of 1–40 μM of AGL and allowed to stand at 37 °C for different time durations of 0.5, 1, and 2 h. Along with this, PBS was used as a negative control in equal volumes of 4% RBC suspension to obtain basal hemolysis. In the positive control, instead of PBS, 1% Triton X-100 was added. Finally, the RBC suspensions in microcentrifuge tubes were subjected to centrifugation for 10 min at 1610*g* and supernatant was taken out to transfer into 96-well plates to estimate the release of hemoglobin content using a microplate reader (Biotek, Cytation 5) at 450 nm. The percentage (%) of hemolysis was calculated using the formula given below:

hemolysis(%)=(test−negativecontrol)/(positivecontrol−negativecontrol)×100%

### 2.17 Light Microscopy

After incubation with a 40 μM concentration of AGL for 2 h, RBC suspensions were subjected to centrifugation at 1610*g* for 10 min to remove supernatant from RBC. The precipitated RBCs were gently resuspended in fresh PBS solution to obtain RBC suspensions. 20 μL of RBC suspension was placed over a microslide, and RBC morphology was recorded under a light microscope (Axio Vert. A1).

### 2.18 Scanning Electron Microscopy (SEM)

RBCs treated with the highest concentrations of 40 μM AGL were precipitated and washed with fresh PBS solution following the same procedure as mentioned under Section 2.17. After that, RBCs were resuspended in 500 μL of 2.5% glutaraldehyde for 30 min for fixation and then centrifuged at 410*g* for 3 min. RBCs were rinsed once with PBS and then with Milli-Q water (18.2 MΩ·cm) three times to remove any fixative and salt traces. Then, immediately RBCs were passed through different grades (30, 50, 70, and 100%) of ethanol each with a 5 min interval.^40^ Finally, 20 μL of RBC suspension in 100% ethanol was placed over the coverslip and proceeded for recording SEM (Sigma, Carl ZEISS) analysis.

### 2.19 Statistical Analysis

All experimental data are presented as mean ± SD/SE, which were derived from three independent experiments each with three replicates of each treatment group (*n* = 9), unless specified otherwise. All the experiments were analyzed in GraphPad Prism 8 version by one-way ANOVA with Dunnett’s or Tukey’s multiple comparison test. *P* values <0.05 were regarded as statistically significant.

## 3 Results and Discussion

### 3.1 Structural Characterization and Purity of Isolated AGL

Confirmation of isolated AGL purity was performed through NMR and HRMS analyses.^41^ The ^1^H NMR (DMSO-*d*_6_, 400 MHz) spectrum of AGL showed shifts at δ 6.64 (1H, dt, *J* = 8.8, 1.8 Hz, H12), 5.75 (1H, d, *J* = 6.1 Hz, OH14), 5.09 (1H, d, *J* = 4.8 Hz, OH3), 4.93 (1H, t, *J* = 6.2 Hz, H14), 4.83 (1H, s, H17A), 4.64 (1H, s, 17B), 4.42 (1H, dd, *J* = 9.8, 6.2 Hz, H15A), 4.16 (1H, dd, *J* = 7.5, 2.8 Hz, OH19), 4.06 (1H, dd, *J* = 10.1, 2.1 Hz, H15B), 3.86 (1H, dd, *J* = 11.0, 2.9 Hz, H19A), 3.29–3.20 (2H, m, H3, H19B), 2.48 (1H, m, H11A), 2.34 (1H, br d, *J* = 13.1 Hz, H7A), 1.99–1.83 (2H, m, H7B, H11B), 1.77–1.60 (5H, m, H1A, H2, H6A, H9), 1.40 (1H, m, H6B), 1.24–1.17 (2H, H1B, H5), 1.10 (3H, s, H18), 0.67 (3H, s, H20) (Figure S1). The ^13^C NMR (DMSO-*d*_6_, 100 MHz) spectra of AGL showed shifts at δ 170.04 (C16), 147.64 (C8), 146.39 (C12), 129.03 (C13), 108.33 (C17), 78.48 (C3), 74.39 (C15), 64.55 (C14), 62.69 (C19), 55.52 (C9), 54.39 (C5), 42.31 (C10), 38.62 (C7), 37.54 (C11), 36.54 (C1), 27.92 (C2), 24.00 (C6), 23.12 (C18), 14.79 (C20). The C4 peak overlapped with the residual solvent signal in the range 40.15–38.90 (Figure S2). HRMS data had calc. mass 351.2171 of AGL with molecular formula C_20_H_31_O_5_ [M + H]+, and we found a mass of 351.2176. The HPLC chromatogram of extracted AGL run through C18 column found a single peak at retention time (*R*_t_) 17.0 min (Figure 1 B).

It was found that purified AGL showed absorption at 225 nm due to π to π* transition of two double bonds as C8–C9 and C11–C12 (Figure S3 A).^42^ The FTIR spectra of AGL exhibited an alcoholic O–H stretching vibration at 3397 cm^–1^ due to the intermolecular hydrogen bond structure and CH_2_ stretching observed at 2927 cm^–1^. The C–O stretching was recorded at 1032 and 1074 cm^–1^ for the primary and secondary alcoholic functions, respectively. The characteristic lactone group responded at 1724 cm^–1^ and stretching vibration at 1416 cm^–1^ due to O–H bonding (Figure S3B).^43,44^ Moreover, XRD patterns showed intense peaks at 2θ = 9.94, 12.12, 14.94, 15.82, 17.72, 18.62, and 22.82° (Figure S3C), indicating high crystallinity of isolated AGL. Morphological analysis of purified AGL by SEM displayed typical crystal blocks of AGL spread across with heterogeneous shapes and sizes having smooth surfaces (Figure S3 D).^45^

### 3.2 AGL Selectively Induces Cytotoxicity in LA7 Cells

The effect of AGL on cell viability was examined in normal HEK-293 and L929 cell lines and the LA7 cell line in a time-dependent fashion by the MTT assay method. Different concentrations of AGL ranging from 1 to 40 μM were treated to cells for up to 72 h. Results demonstrated that AGL at highest concentrations of 40 μM did not have (>80% cell viability) cytotoxic effects on normal HEK-293 and L929 cell lines (Figure 1C,D). However, AGL causes dose-dependent inhibitory effects on the LA7 cell proliferation (Figure 1E and Figure S4). The similar pattern was also observed with increasing time durations from 24 to 72 h. The IC_50_ values of AGL on LA7 were 19 ± 2.5 μM for 24 h, 12 ± 1.9 μM for 48 h, and 10 ± 0.8 μM for 72 h (Figure 1 F–H). Similar results using AGL are reported earlier in the literatures against BC.^31,46^ These results suggested that AGL possesses specific inhibitory effects to LA7 cells, indicating its potential in cancer therapy.

### 3.3 AGL Inhibits the Formation of Single LA7 Cells to Form Colonies

The inhibitory effects of AGL on the colony-forming efficiency of LA7 cells were assessed via the colony formation assay.^30^ The colony-forming cells were treated with AGL of 10, 20, and 30 μM concentrations for a 24 h duration. It was found that AGL inhibited the ability of LA7 to form colonies after 10 days of culture (Figure 2A). The percentage of number of colonies significantly reduced from 31 to 2% with 10 to 30 μM of AGL treatment. Moreover, very negligible colonies were observed after 30 μM of AGL treatment, which was comparable with a similar concentration of an FDA-approved drug, TAM, for the treatment of BC.^47^ Taken together, these data indicate the considerable antiproliferative activity of AGL on LA7 cell colony formation. It was reported that a single LA7 cell has the capacity to divide continuously for 63 passages and form a single mammosphere containing ductal epithelial, myoepithelial, and alveolar cell lineage.^48^ LA7 cells expressed various markers including K-14, K-18, β-casein, p63, E-cadherin, CD44, and CD29, which are key regulators in the maintenance of LA7 cells to form heterogeneous mixtures of colonies.^5^ In addition, the expressions of OCT4, SOX-2, Nanog, and c-Myc transcription factors were also associated with the initiation of LA7 cells' growth and survival.^49^ AGL may interact with these subcellular proteins to suppress LA7 cells for the formation of colonies.

**Figure 2:**
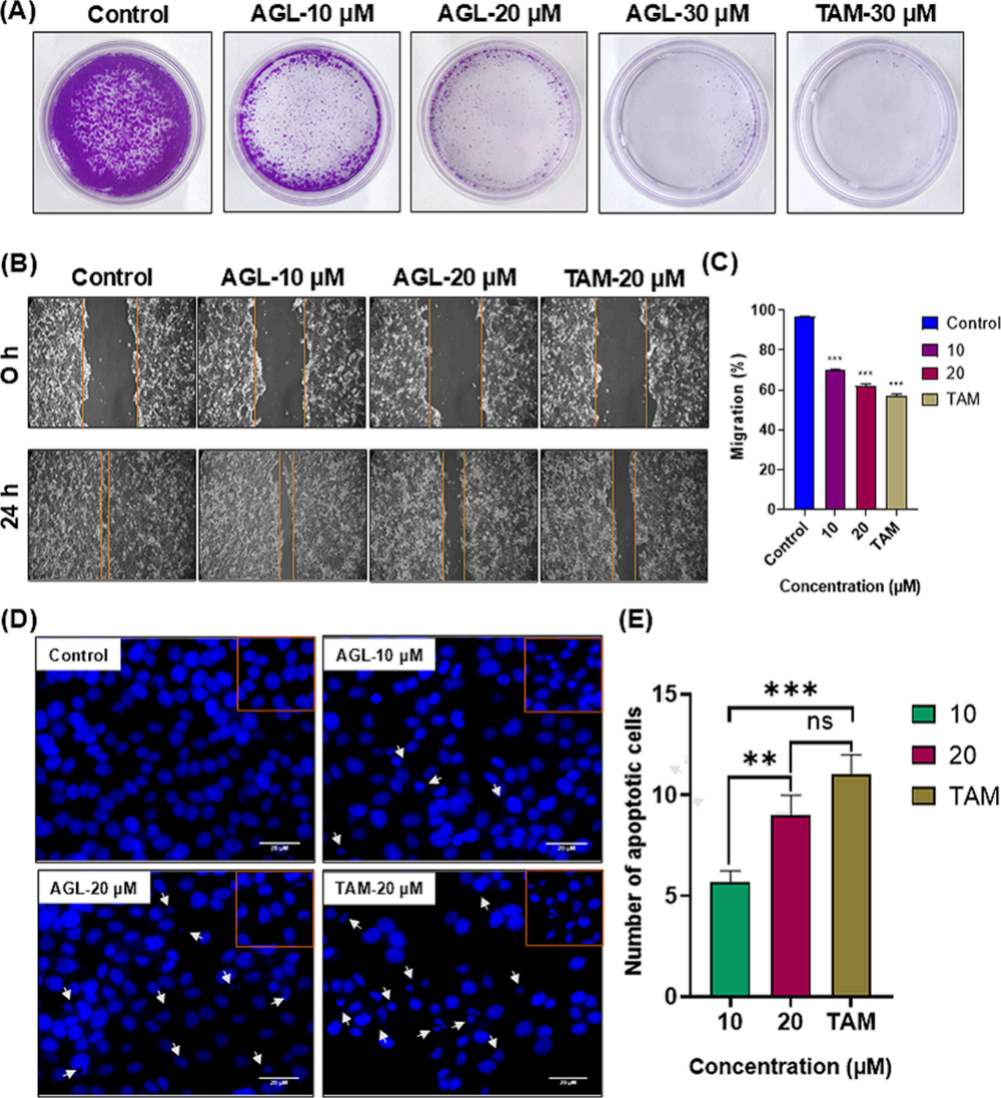
(A) Inhibitory role of AGL (10–30 μM) along with TAM (30 μM) on LA7 cell colony formation after 10 days of treatment. (B) *In vitro* cell migration assay to measure the effects of AGL on migration and mobility of LA7 cells. The migration gaps of LA7 cells after 24 h of treatment with AGL and TAM. (C) Bar graph showing the migration rates (%) of each group after 24 h of treatment. Statistical data was presented between control and treated groups, which were analyzed by one-way ANOVA followed by Dunnett’s test (***P < 0.001, **P < 0.01, *P < 0.05, ^ns^ *P* > 0.05). The effects of AGL on nuclear morphology analyzed using DAPI staining. (D) Nuclear morphological images of LA7 cells after treatment with untreated control, AGL (10–20 μM), and TAM (20 μM) for 24 h. (E) Bar graph presenting the dose-dependent apoptotic cells numbers as compared to control. Nuclear changes induced by AGL and TAM are indicated by the white arrow. Data presented are mean of three independent experiments with SD (*n* = 9). Scale bar 20 μM. Statistical analysis between groups was analyzed by Tukey’s one-way ANOVA (***P < 0.001, **P < 0.01, *P < 0.05, ^ns^ *P* > 0.05).

### 3.4 AGL Prevents Cellular Migration of LA7 Cells

Cell migration following creation of a scratch in cell monolayer is a useful technique to investigate the effects of drug on cell migration, mobility, cell–cell interaction, and invasiveness of cancer cells.^50^ For this, confluent monolayer LA7 cells with scratches were made in the center and incubated with 10 and 20 μM of AGL for 24 h. From microscopy imaging, it was demonstrated that AGL delayed wound gap closure or the migration ability of LA7 cells with increasing concentrations compared to untreated control (Figure 2B). It was observed that a very smaller number of cells were moved to the scratch site with cell migration rates of 69 and 61% at 10 and 20 μM of AGL, respectively (Figure 2C). Earlier report stated that the effects could be due to inhibition of NF-κB-THOC1 proteins, which are involved in malignancy and stemness of cancer.^6^

### 3.5 AGL Induces DNA Damage in LA7 Cells

Nuclear staining using DAPI was carried out to investigate the cytotoxic effects of AGL on the nuclear integrity of LA7 cells. It was found that AGL treatment for 24 h in LA7 cells resulted in chromatin condensation, membrane blebbing, and irregular edges around the nucleus, which are the distinctive characteristics of apoptotic nuclei.^36^ The nuclei of untreated cells were observed to be round, smooth and uniform with clear edges (Figure 2D). The average numbers of apoptotic cells at 10 and 20 μM were 6 and 9, respectively. The results revealed increased number of apoptotic cells from 10 to 20 μM of AGL treatment (Figure 2E). Moreover, nuclear morphological findings also suggested that AGL, being as effective as the known therapeutic drug TAM, indicates its potential as BC therapy. Genomic instability and fragmentation of DNA is the hallmark of apoptotic cell death.^51^ This result indicates that AGL restrains aberrant proliferation of cells and induces DNA fragmentation, resulting in apoptosis in LA7 cells.

### 3.6 AGL Disrupts the Mitochondrial Membrane Integrity of LA7 Cells

MMP is essential for maintaining the homeostasis of mitochondrial metabolism and membrane-driven ATP synthesis. Decreased MMP or depolarization of MMP is an indicator of unhealthy conditions of cellular machinery.^52,53^ During apoptosis, the mitochondrial membrane integrity becomes disrupted or depolarized, which can be analyzed by cationic dye, JC-1. The JC-1 dye has a tendency to enter the mitochondria, become aggregated, and emit red fluorescence in healthy cells, whereas it presents in the cytoplasm in monomeric form in apoptotic cells and emits green fluorescence. The degree of green to red fluorescence intensity indicates the state of mitochondrial membrane integrity. It was observed that in untreated control cells, only bright-red fluorescence was observed while AGL promoted the dose-dependent increment of green/red fluorescence intensity, a clear indication of mitochondrial membrane disruption in LA7 cells (Figure 3A). Mitochondria contain a family of proteases that are regulators of caspases involved in apoptotic cell death pathways. Studies have suggested that mitochondrial membrane damage is correlated with the initiation of early apoptosis.^54,56^ Alteration of MMP indicates disruption of mitochondrial structure and function which leads to cytochrome C (Cyt) release into the cytosol and facilitates apoptosis.^55^ Earlier investigation showed that AGL could trigger the Cyt C translocation from mitochondria to cytoplasm in MDA-MB-231 cells.^17^ This current study also revealed that AGL disrupts mitochondrial membrane potential and might induce LA7 cell death via the Cyt C-mediated pathway.

**Figure 3:**
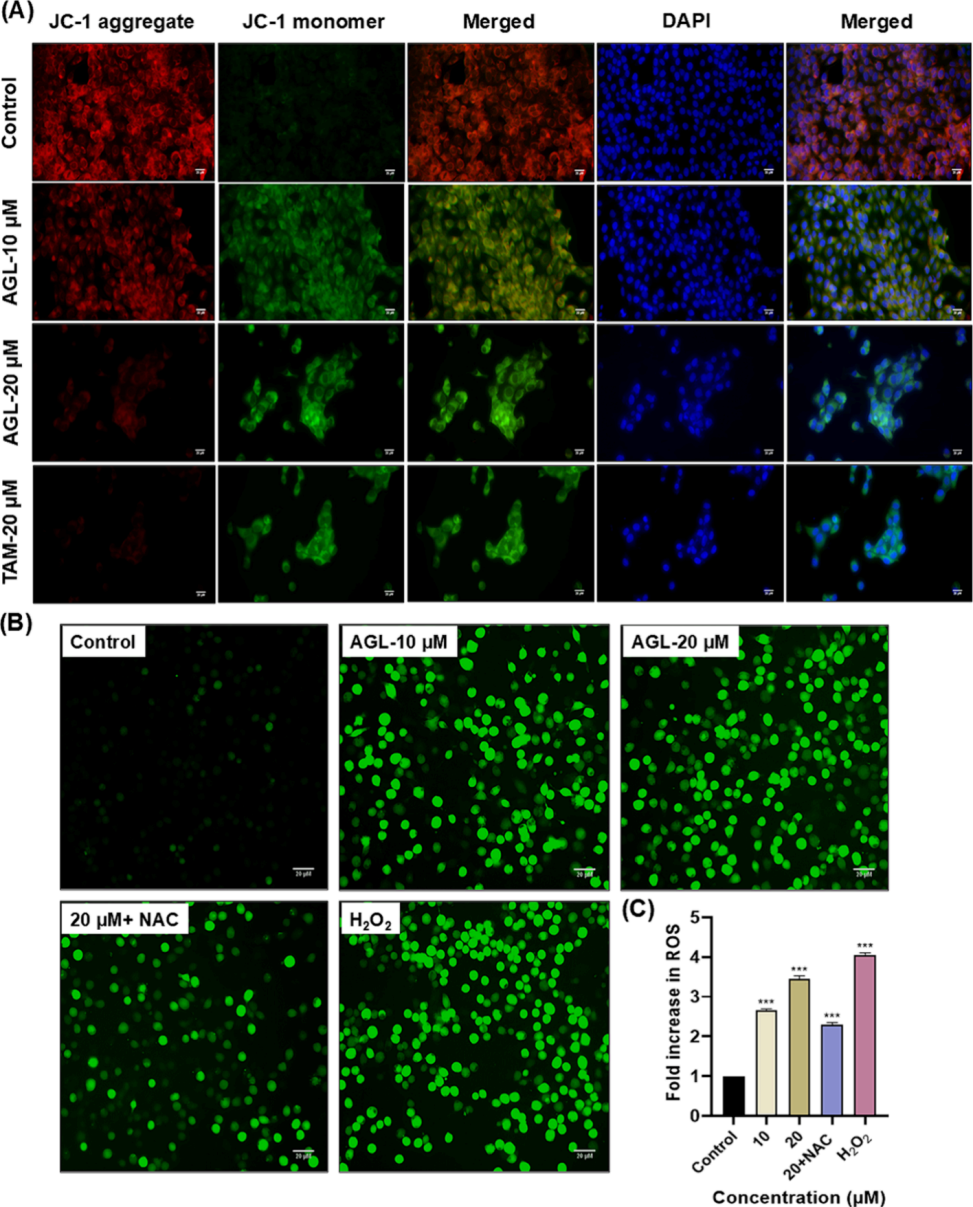
(A) AGL-induced mitochondrial membrane potential analysis in LA7 cells. AGL (10 and 20 μM) and TAM (20 μM) showed disruption of MMP of LA7 cells. Cell nuclei were counterstained with DAPI dye. (B) Determination of intracellular ROS production after 24 h treatment in LA7 cells using the DCFH-DA method. Cells were treated with control (PBS), AGL (10–20 μM), NAC (100 μM), and H_2_O_2_ (100 μM). AGL-induced production of ROS in LA7 cells. (C) Quantification of intracellular ROS production was analyzed by a fluorescence microplate reader. Data shown are mean of three independent experiments with SD (*n* = 9). Scale bar 20 μM. Statistical analysis between control and treatment groups was performed by Dunnett’s one-way ANOVA (***P < 0.001).

### 3.7 AGL Promotes Generation of Intracellular ROS in LA7 Cells

Disruption of MMP is directly associated with the alteration of cellular biochemical processes, such as intracellular ROS generation. To investigate the role of AGL on the generation of oxidative stress in cancer cells, a cell-permeant fluorescent dye DCFH-DA was used. Once inside the cells, the dye deacetylated into a nonfluorescent compound, which is turned into oxidized fluorescent 2′,7′-dichlorofluorescein (DCF) upon cellular ROS generation. An excessive ROS generation is directly correlated with the activation of apoptosis signaling pathways.^54^ ^,56^ Classically, most of the anticancer drugs trigger cancer cell apoptosis via the ROS-mediated pathway.^57,58^ It was observed that treatment with 10 and 20 μM AGL in LA7 cells causes significant enhancement of ROS in terms of DCF fluorescent intensity. The pretreatment of 100 μM NAC resulted in a decrement of ROS level, further substantiating our findings of AGL-induced ROS generation in cancer cells (Figure 3B,C). As mitochondrial damage is linked with alteration of MMP and generation of oxidative stress, it playes a crucial role in the apoptosis induction.^59^ These results confirmed that AGL significantly promotes MMP loss and high ROS level in LA7 cells in a dose-dependent manner. Studies have reported that AGL induces ROS/JNK signaling pathways or ER stress-mediated apoptosis through intracellular ROS generation in cancer cells.^60,61^ AGL may also interact with these mechanisms to inhibit LA7 cell proliferation. Moreover, AGL also likely to play a protective role in other pathological conditions via maintaining oxidative/antioxidative balance. Recent studies demonstrated that AGL decreases ROS generation via regulating the Nrf2/HO-1 pathway against ulcerative colitis or as a cerebroprotective role through regulating the reactive nitrogen species (RNS) formation.^62,63^ Therefore, AGL can play a critical role for ROS-induced cancer cell death while protecting normal cells from oxidative damage.

### 3.8 AGL Induces Apoptosis in LA7 Cells

The AO-PI double staining was performed to observe nuclear morphological changes during different stages of apoptosis. AO stains both live and membrane-compromised cells and emits green color, while PI only stains membrane-compromised or membrane integrity-lost dead cells and emits red color.^64^ It was observed that after 24 h, untreated control cells showed a uniform green-stained nucleus representing intact viable cells. While it was observed that with increasing concentrations from 10 to 20 μM of AGL, the number of red fluorescent membrane-compromised dead or dying cells significantly increased in LA7 cell lines. Early or moderate apoptotic cells featured with bright-green fluorescence due to chromatin condensation and membrane blebbing, and fragmentation with a reddish-orange color was observed (Figure 4 A). Apoptosis and necrosis are two major forms of cell death differentiated by swelling of necrotic cells while apoptotic cells undergo shrinkage.^65^ The AO-PI double staining demonstrated morphological evidence of early and late apoptotic cell death with AGL treatment. This finding supports the fact that AGL induces apoptosis rather than necrosis in LA7 cells. In the proliferation, cell survival, and aberrant migration of cancer, various signaling pathways, namely, PI3K/AKT/mTOR, MAPK/ERK, and NF-κB, were involved.^66,67^ Moreover, different transcription factors and regulatory proteins also play a crucial role in downstream or upstream activation of signaling pathways, protection of DNA damage, and telomere dysfunction in cancer cells.^68^ A study has reported that nuclear factor related to kappaB binding protein (NFRKB), a regulatory subunit G of the INO80 complex, is involved in the protection of telomere degradation, which is highly expressed during cancer.^69^ Therefore, an in-depth interaction between AGL with targeted proteins or investigation of pathway inhibition driving apoptosis of LA7 cells is further needed in the designing of effective therapeutic strategies against BC.

**Figure 4:**
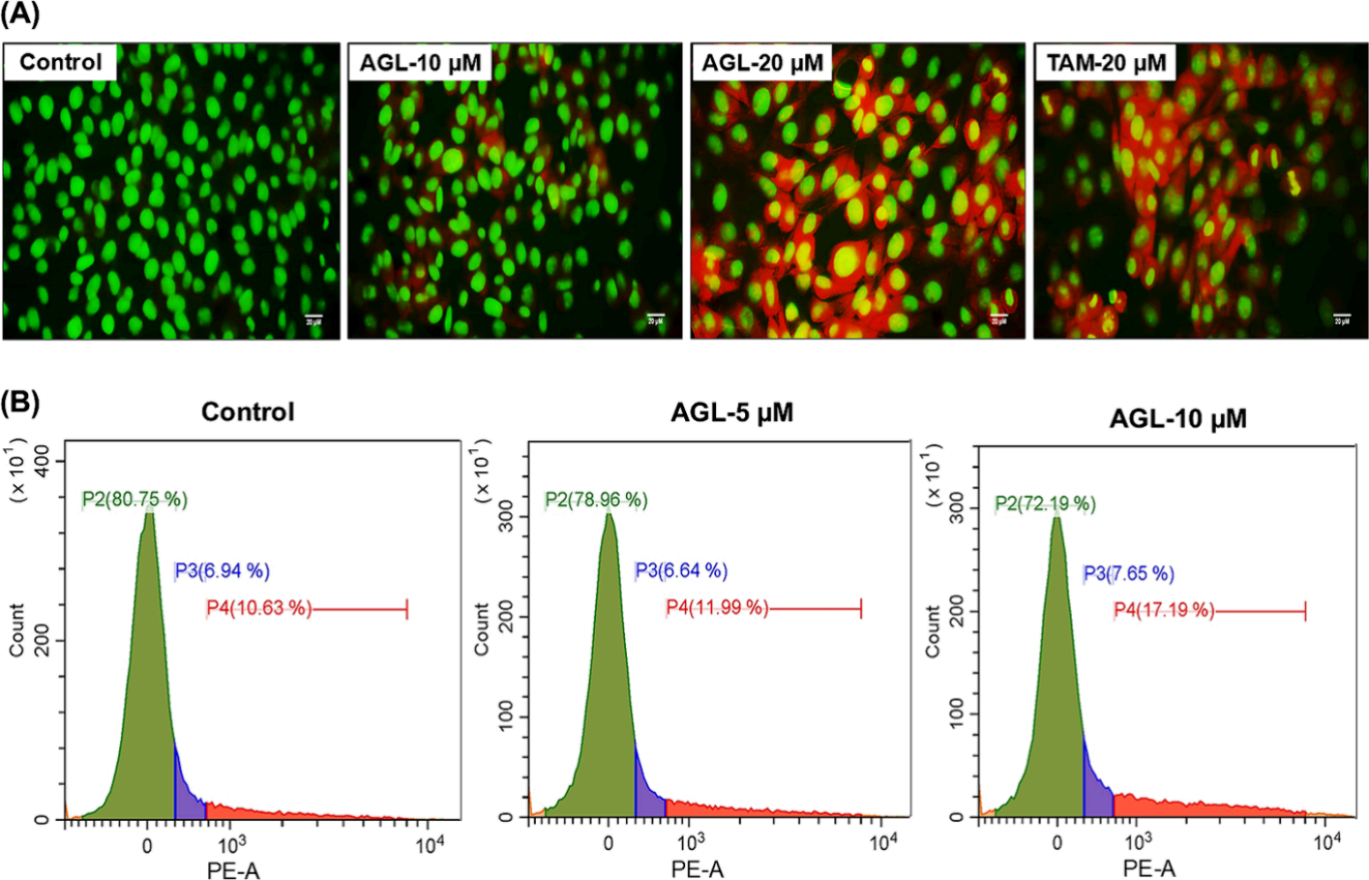
(A) Fluorescence microscopy image of AO-PI dual staining in LA7 cells after 24 h. Untreated control cells have a normal structure with no prominent apoptosis. Cells treated with 10 μM AGL demonstrated early to moderate apoptotic features. Late apoptosis event observed at a concentration of 20 μM for both AGL and TAM. Scale bar: 20 μM. (B) AGL induced cell cycle arrest in the LA7 cells. Cells were treated with 5 and 10 μM of AGL for 24 h, and cell cycle progression inhibition was assessed by flow cytometry using PI staining.

### 3.9 AGL Induces Cell Cycle Arrest in LA7 Cells

One of the crucial strategies to halt cancer cell progression is to induce cell cycle arrest.^70^ Most of the anticancer drugs exert an inhibitory effect on cancer cell progression by targeting cell cycle checkpoints.^71^ Therefore, to investigate AGL inhibitory effects of DNA intercalating dye, PI was used to quantify a relative amount of DNA in different G0/G1, S, and G2/M phases of the cell cycle. Increasing concentrations of AGL retarded the progression of the LA7 cell cycle at the G2/M phase (Figure 4B). The cell percentage (%) at the G2/M phase increased from 10.63% in untreated control to 11.99 and 17.19% at 5 and 10 μM in AGL, respectively, suggesting the fact that LA7 cells were undergoing apoptosis with G2/M phase arrest. To date, one study has reported that AGL induces G2/M cell cycle arrest in human MDA-MB-231 breast cancer cells, while our finding also showed the similar effect of AGL in rat LA7 cells.^59^

DNA damage response serves as a critical signal associated with ROS-induced apoptotic cell death and mediates anticancer effects in most of the anticancer drugs.^72^ Recent studies have reported that the inhibition of cell cycle progression at the G2/M phase induces accumulation of excessive mitochondria and generation of mitochondrial superoxide followed by impairment of mitochondrial homeostasis.^73^ Another study also stated that prolonging cell cycle arrest may allow production of mitochondrial ROS that eventually inflicts DNA damage in cancer cells.^74^ Therefore, DNA damage, loss of MMP, and excessive intracellular ROS generation support the observations that AGL promotes inhibits LA7 cells and to undergo apoptosis along with the undergoing cell cycle arrest at the G2/M phase.

### 3.10 Identification of Possible AGL Targets Using Computational Studies

#### 3.10.1 Identification of Possible Anticancer Targets for AGL

The SwissTargetPrediction, Pass Analysis, and DIGEP databases were used for identifying the possible targets of AGL involved in inhibition of LA7 cells. The top-scored targets from each method were identified and are tabulated in Figure 5. Additionally, the drug targets already reported from LA7 cells were identified through literature mining. Among these proteins, we considered four proteins, namely, ER-β, BCL-2, NF-κB, and PKC-α, as possible targets of LA7 cells for further analysis.

**Figure 5:**
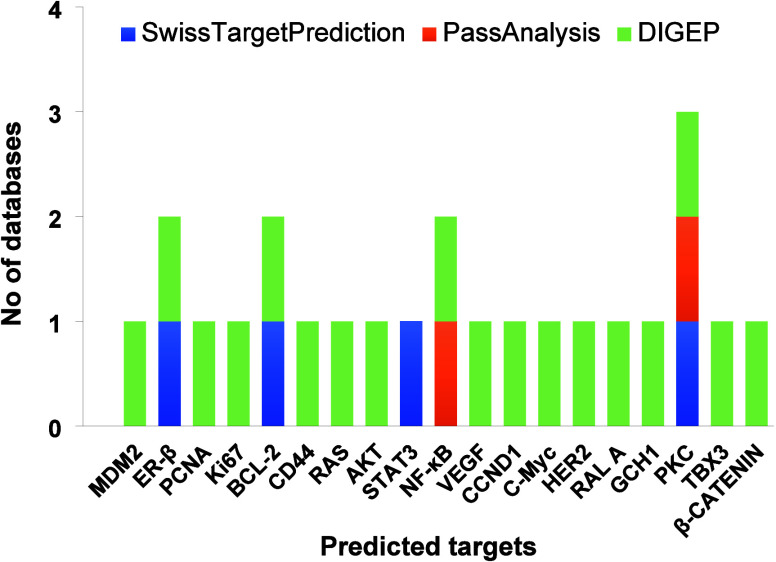
Target identifications of AGL using SwissTargetPrediction, Pass Analysis, DIGEP databases, and literature studies.

#### 3.10.2 Molecular Docking Analysis

Molecular docking was performed in AutoDock 4.2 to study the binding mode of AGL in the active site of four selected proteins, namely, ER-β, BCL-2, NF-κB, and PKC-α. Except for NF-κB, the remaining three PDB structures have the cocrystallized ligand. Thus, the active site was considered 5 Å within the binding region of the cocrystallized ligand. The active site of NF-κB was identified using the CASTp server (http://sts.bioe.uic.edu/castp/index.html?1nfi). Among the four selected targets, AGL shows poor binding affinity toward ER-β (binding energy: 14.23 kcal/mol). Thus, we omitted ER-β from our study, and the remaining three proteins were considered for further analysis. Table 1 represents the binding energy and interacting amino acids of AGL in the active sites of three proteins. The results show that AGL bound well within the active site of three selected proteins. The binding energy ranged from −6.69 to −7.89 kcal/mol, and it was further confirmed that AGL is able to bind well within the active site of three selected proteins. The interaction of AGL with the different amino acid residues of target proteins including BCL-2, NF- κB, and PKC-α is depicted in Figure 6.

**Table 1:** Molecular Docking Results of the AGL in the Selected Three Proteins

targets	binding energy(kcal/mol)	ligand efficiency(kcal/mol)	inhibitory constant	interacting amino acids
BCL-2 (PDB: 4LVT)	–6.69	–0.27	12.44 μM	Gly142, Leu198
NF-κB (PDB: 1NFI)	–6.88	–0.28	9.09 μM	Gly31, Ser45 and His58
PKC-α (PDB: 3IW4)	–7.89	–0.32	1.66 μM	Leu345, Phe350, Lys368, Glu387

**Figure 6:**
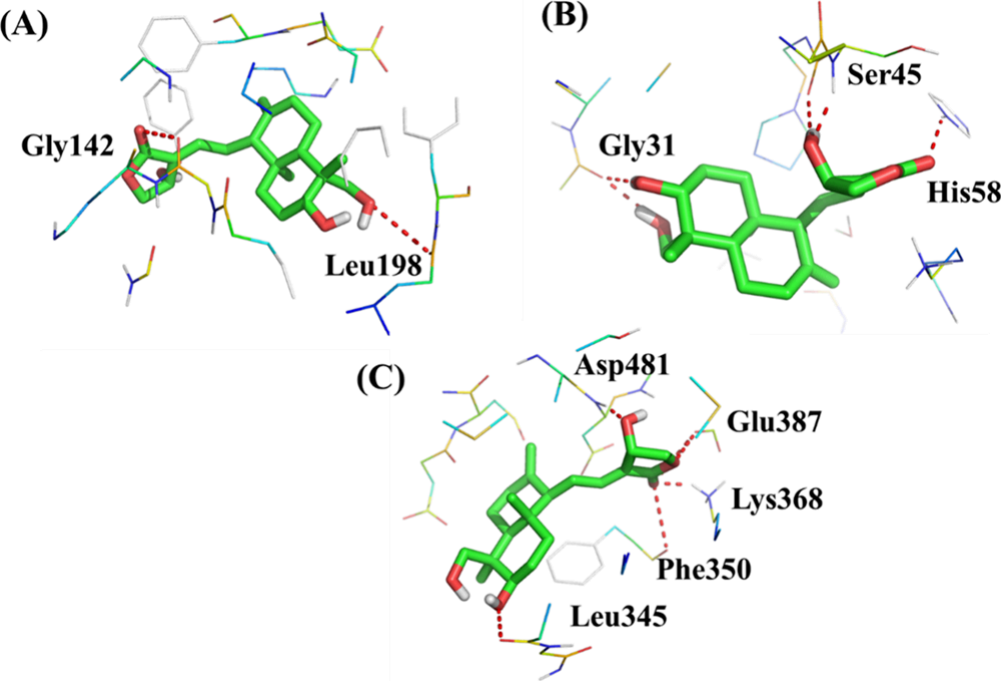
AGL in the active sites of (A) BCL-2, (B) NF- κB, and (C) PKC-α.

#### 3.10.3 MD Simulation Analysis

A 100 ns MD simulation was performed on each protein–ligand complex using Gromacs Version 2021.4 to evaluate the AGL stability in the three protein active sites. The MD simulation results are depicted in Figure 7. Figure 7A represents the protein backbone RMSD. The results suggested that BCL-2 and NF-κB exhibited a similar type of distribution, and PKC-α showed larger fluctuation initially and stabilized after 20 ns. However, overall, the results showed that the backbone RMSD fluctuates between 0.1 and 0.3 nm, which showed that the protein does not fluctuate too much throughout the 100 ns simulation period. This is further confirmed by analyzing the ligand RMSD. The ligand RMSD results showed that the fluctuation of AGL is between 0.025 and 0.17 nm (Figure 7B). These findings show that the AGL bound well within the active site of three proteins. The total number of hydrogen bonds formed between AGL and protein targets were further supported the results. On average, AGL was able to maintain 3–5 H bonds in the three proteins throughout the simulation period (Figure 7C). These results support that the AGL helps in stabilizing these three proteins, and it may be a potential candidate for anticancer treatment especially to BC.

**Figure 7:**
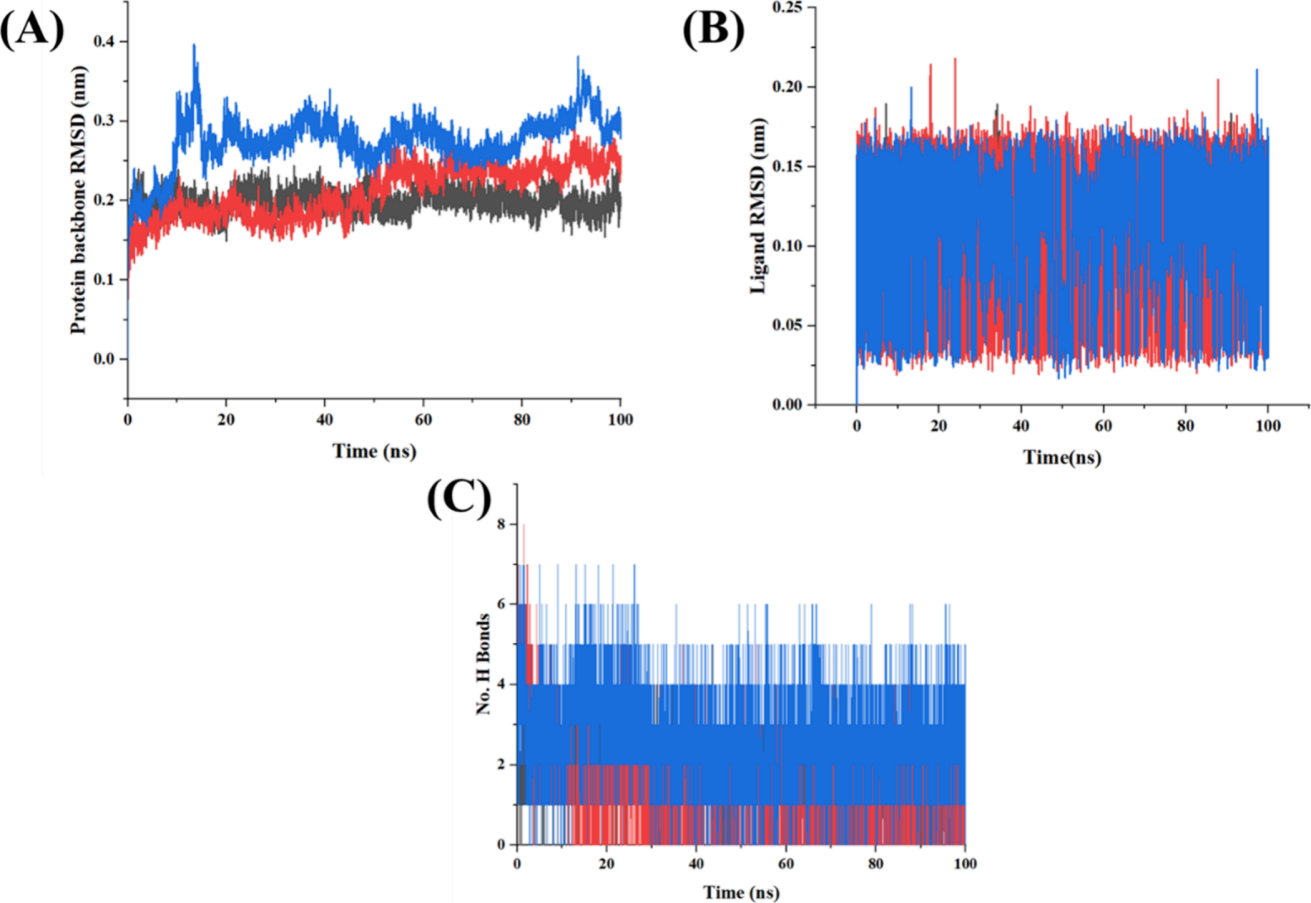
MD simulation results including (A) protein backbone RMSD, (B) ligand RMSD, and (C) no. of H bonds of AGL in the active site of BCL-2 (black), NF-κB (red), and PKC-α (blue) respectively.

Additionally, the binding energies of AGL with the respective protein targets was calculated using the MM/PBSA method. The results show that the binding energies ranged from −32.40 to −35.80 kcal/mol (Table 2). These results suggest that AGL has good binding affinity with the selected targets.

**Table 2:** Binding Free Energy Calculation Results of AGL in Three Selected Protein Targets. The values represent mean±SD.

proteins	ΔVDWAAS (kcal/mol)	ΔEEL (kcal/mol)	ΔEGB (kcal/mol)	ΔESURF (kcal/mol)	–*T*Δ*S*(kcal/mol)	ΔG (kcal/mol)
BCL-2	–55.92 ± 2.40	–9.35 ± 1.70	32.75 ± 1.80	–7.10 ± 0.25	3.85 ± 1.20	–35.80 ± 3.00
NF-κB	–57.20 ± 2.50	–98.80 ± 6.00	117.90 ± 5.10	–7.65 ± 0.18	9.95 ± 3.60	–35.00 ± 4.10
PKC-α	–53.45 ± 2.20	–4.50 ± 2.15	28.40 ± 2.00	–6.70 ± 0.22	3.45 ± 1.40	–32.40 ± 2.75

Earlier studies have also reported the beneficial role of these proteins in the initiation, growth, and metastasis of cancer. NF-κB is a pro-inflammatory transcription factor (TF) that transactivates genes associated with cell growth, survival, and metastasis and modulates a network of genes that underlie carcinogenesis.^75^ Study has reported that around 75% initial BC cases show overexpression of BCL-2, which is activated by NF-κB.^76^ PKC are a class of serine–threonine kinases that play a pivotal role in the regulation of diverse biological function and prevent cancer cells from undergoing apoptosis. It was reported that mitochondrial PKC-α phosphorylates antiapoptotic protein BCL-2 to get activated resulting in increased cell survival and therapeutic resistance.^77^ Molecular profiling of LA7 cells showed that BCL-2 and NF-κB were expressed in LA7 cells to maintain the cell survival and abrogation of the death signal pathway. Notably, it was observed that the expression level of antiapoptotic BCL-2 protein was significantly higher in LA7 cells as compared to other mammary breast cancers including MDA-MB-231, SKBR3, and RBA cells.^78^ Moreover, LA7 cells also expressed PKC-α, which might be involved in the activation of pro-survival pathways in LA7 cells.^79,80^ These studies confirmed the inhibitory role of AGL against LA7 cell proliferation via targeting BCL-2 and PKC-α proteins or NF-κB signaling pathways. Furthermore, SAR studies of AGL structure in the position of lactone ring, conjugated double bonds, and functional groups or investigation of combination therapies with other chemotherapeutic drugs, including tamoxifen, doxorubicin, or fulvestrant, will be required to improve its activity and overcome drug resistance during BC therapy.

#### 3.10.4 ADMET Profiling

The ADMET properties of the AGL were evaluated using the online server ADMETlab 3.0 (https://admetlab3.scbdd.com/). The results showed that the majority of the properties, including molecular weight, number of hydrogen bond donors/acceptors, number of rotatable bonds, Quantitative Estimation of Drug likeness (QED), and Lipinski’s rule of five, are in the acceptable range. Table 3 displays the ADMET properties of AGL. This further confirms that AGL may not show any systemic toxicity and has the potential to emerge as an anticancer agent.

**Table 3:** Predicted ADMET Properties of AGL^a^

properties	value	acceptable range
molecular weight	350.21	100–600
nHA	5.0	0–12
nHD	3.0	0–7
nRot	3.0	0–11
TPSA	86.99	0–140
logS	–4.259	the logarithm of aqueous solubility value
logP	2.482	the logarithm of *n*-octanol/water distribution coefficient at pH = 7.4
logD	2.766	the logarithm of *n*-octanol/water distribution coefficient
QED	0.411	attractive: >0.67; unattractive: 0.49–0.67; too complex: <0.34
Lipinski rule	0.0	MW ≤500; logP ≤5; Hacc ≤10; Hdon ≤5
Caco-2 permeability	–5.348	>−5.15 log unit
HIA	0.002	category 1: HIA+(HIA <30%); category 0: HIA–(HIA ≥30%)

a nHA: number of hydrogen bond acceptors; nHD: number of hydrogen bond donors; nRot: number of rotatable bonds; TPSA: topological polar surface area; HIA: human intestinal absorption.

### 3.11 Nonhemolytic Effects of AGL

Drug-induced hemolysis is one of the major complications associated with the chemotherapy agents.^81^ Moreover, RBCs are the most abundant cell type that come into contact with drug molecules following intravenous injection into the blood circulation system. An unfavorable interaction between drug molecules and RBCs may lead to release of intracellular hemoglobin, which eventually indicates its toxicity.^23^ Therefore, any proposed drug candidate must be devoid of hemolytic activity to prevent the further exacerbation of the cancer pathophysiology. It was found that AGL did not cause hemolysis to SD rats and human RBCs with increasing concentrations and incubation time. The hemolysis rate of AGL at 40 μM concentration was found to be <5% (Figure 8). As per ISO 10993-4 guidelines, the hemolysis rate of <5% can be considered as a hemocompatible agent.^82^ These results indicate that AGL did not lyse the RBC membrane structure and will not cause any side effects during BC therapy. However, further comparative studies with other established chemotherapeutic drugs like paclitaxel or doxorubicin or platinum-based cytotoxic drugs will be required to understand better safety of AGL.

**Figure 8:**
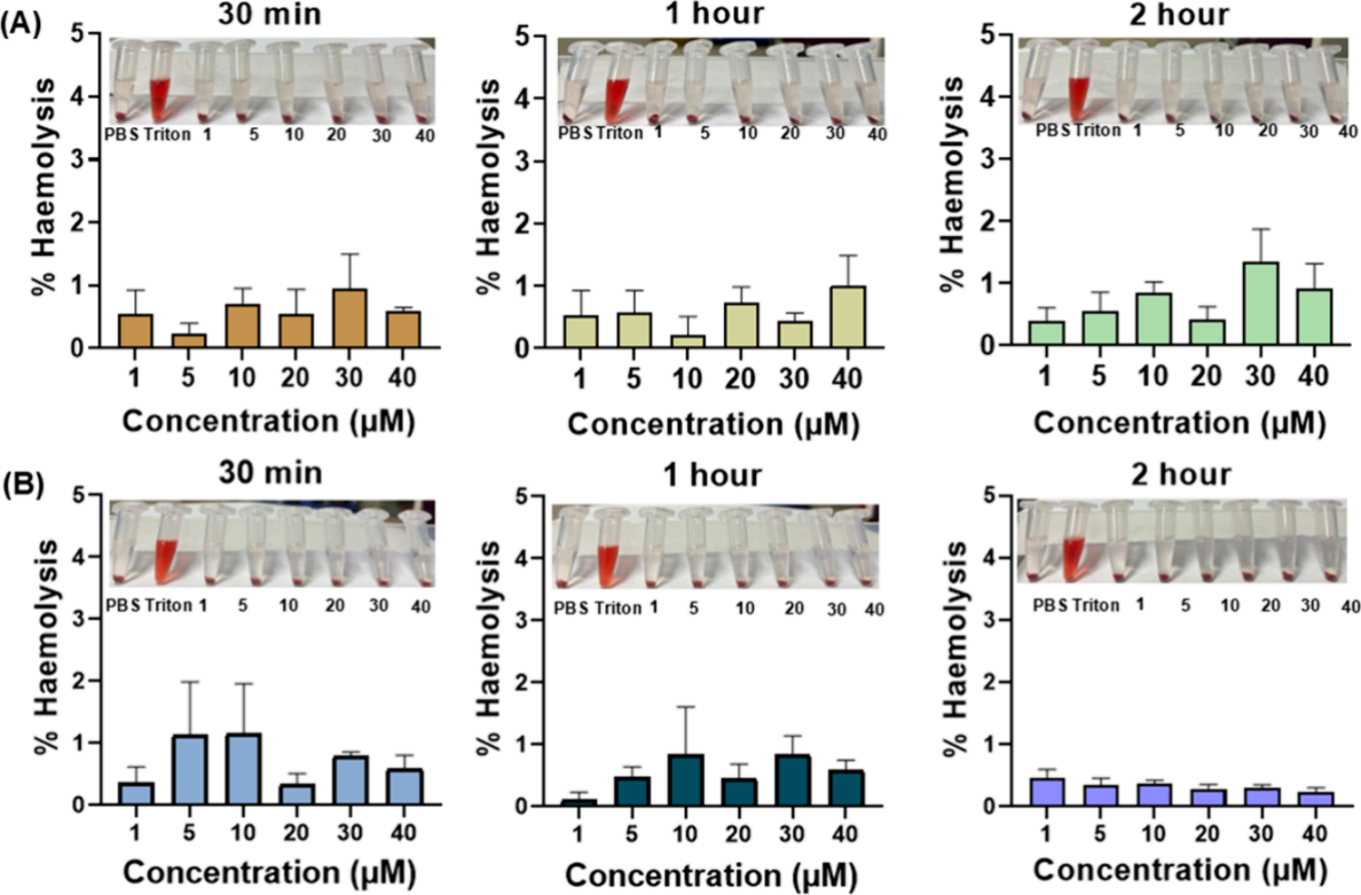
Hemocompatibility study of AGL at different time points. The percentage (%) of hemolysis rate after various concentrations of AGL treatment using (A) SD rats and (B) human blood samples. Inset: photographs of RBC dispersions treated with PBS, Triton-X-100 (1%), and AGL (1–40 μM) solutions. Data presented are the mean of three independent experiments with SE (*n* = 9).

### 3.12 AGL Did Not Alter the RBC Morphology

The light microscopy and SEM study were performed to further confirm the results of hemocompatibility analysis of AGL in SD rats and human blood samples. Morphological images of light microscopy showed that at 40 μM AGL, the RBCs retained their typical biconcave shape, similar to the untreated control. The treatment with Triton X-100 (0.5%), a nonionic detergent, causes swollen spherocyte shapes of RBCs due to intercalation with the lipid bilayer, increased lytic volume, and disruption of the membrane structure.^83,84^ A magnified view of RBCs' morphology by the SEM study further confirmed the findings obtained by light microscopy. After incubation with 40 μM AGL, no visible membrane deformation was observed in SD rats and human RBC structure. In both control and AGL-treated groups, RBCs were found to be normally biconcave disc in shape. In Triton X-100-treated groups, it was clearly observed that the RBC membrane was significantly destroyed, which results in RBC lysis (Figure 9). These findings further confirmed that AGL is nonhemolytic in nature and will not cause any side effects during BC therapy.

**Figure 9:**
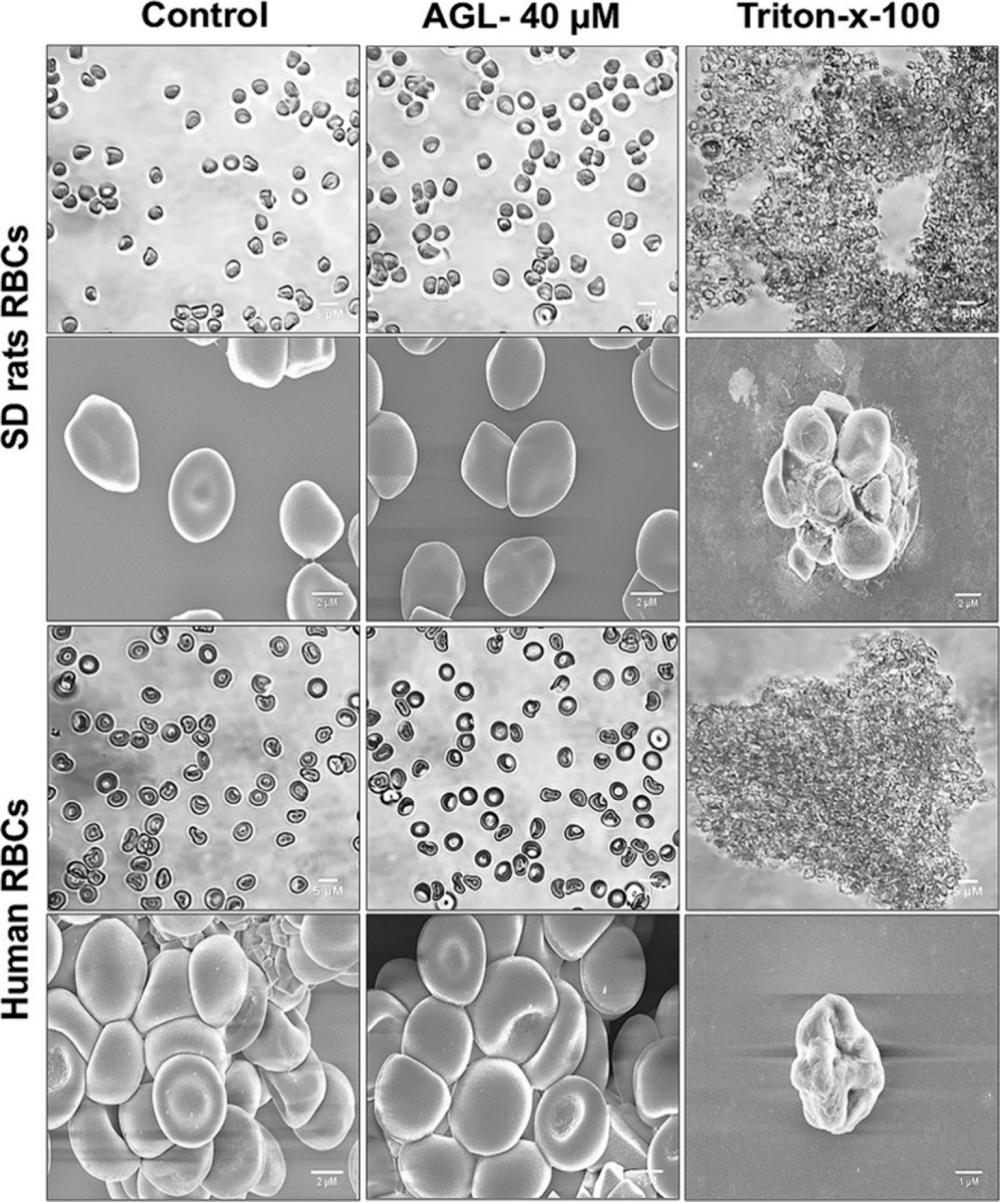
Morphological observation of RBCs by light microscopy and scanning electron microscopy. AGL (40 μM) causes no changes in SD rats and human RBC structure as compared to the untreated control.

## 4 Conclusions

In this study, a simple, rapid, cost-effective solvent extraction-gravimetric column chromatography technique is demonstrated to isolate AGL from *A. paniculata* plant leaf extract. In LA7 cells, AGL showed antiproliferative effects via induction of DNA damage, disruption of mitochondrial membrane integrity, generation of oxidative stress, and cell cycle arrest at the G2/M phase. *In silico* findings further revealed that AGL can interact with hallmark proteins involved in anticancer treatment, namely, BCL-2, NF-κB, and PKC-α, as possible targets for inhibition of LA7 cell progression and induction of apoptosis. Furthermore, our results showed that AGL is nonhemolytic in nature as it induced negligible RBC lysis (<1%) of SD rat and human blood with no visible alteration of the RBC morphology. Taken together, our study presents a novel approach to extract AGL from plants, which could be investigated as a safe and potent anticancer lead for breast cancer therapy.

These findings establish a valuable methodology for isolation of pure natural phytomolecules with the possibility of affordable scale-up production for pharmaceutical application. Hemolytic toxicity is one of the key limitations of several marketed anticancer drugs, and therefore, AGL has promising potential, which can be investigated through comparative studies with existing anticancer drugs for efficacy and safety. However, lack of further in-depth invetigations of underlying molecular signaling pathways, *in vivo* efficacy of AGL in LA7 cell-transplanted syngeneic tumor model, and detailed systemic toxicity profile in animal models are limitations of the current study, which are necessary before making any effort for translating AGL as a potential agent for breast cancer therapy.

## Supplementary Material



## Notes

^1^H NMR spectra of isolated AGL (Figure S1), ^13^C NMR spectra of isolated AGL (Figure S2), physico-chemical characterizations of isolated AGL (Figure S3A–D), effects of AGL on LA7 rat mammary tumor cells cytotoxicity analyzed by MTT assay (Figure S4), and list of identified LA7-specific gene expression data sets from literature mining (Table S1) (PDF)

## Abbreviations

BC: breast cancer
DMBA: 7,12-dimethylbenz­[*a*]­anthracene
RBCs: red blood cells
TLC: thin layer chromatography
NMR: nuclear magnetic resonance
HRMS: high-resolution mass spectrometry
DAPI: 4′,6-diamidino-2-phenylindole
JC-1: 5,5,6,6′-tetrachloro-1,1′,3,3′ tetraethylbenzimi-dazoylcarbocyanine iodide
MMP: mitochondrial membrane potential
ROS: reactive oxygen species
NAC: *N*-acetyl cysteine
DCFH-DA: 2′,7′-dichlorodihydrofluorescein diacetate
AO: acridine orange
PI: propidium iodide
