# Supplementary material for: Andrographolide Selectively Inhibits the Growth of LA7 Mammary Adenocarcinoma Cells
Source: ACS Omega. 2025 Nov 20;10(47):57052–66. doi: 10.1021/acsomega.5c04617 (PMC12676339; doi:10.1021/acsomega.5c04617)
Supplement: Supplementary file 1 [file ao5c04617_si_001.pdf]

Supporting Information

Andrographolide selectively inhibits the growth of LA7 mammary adenocarcinoma cells

Kallol Roy[1, 4], Parthiv Kar[1, 4], Saikat Haldar[1, 4], Sarangthem Dinamani Singh[2, 4], Selvaraman Nagamani[2, 4], Himangsu K Bora[1, 4], Binoy K Saikia[3, 4], Rituraj Konwar[1, 4*]

[1]Centre for Preclinical Studies (CPS), Biological Sciences and Technology Division (BSTD), CSIR-North East Institute of Science and Technology (CSIR-NEIST), Jorhat 785006, Assam, India

[2]Advanced Computation and Data Sciences Division (ACDSD), CSIR-North East Institute of Science and Technology (CSIR-NEIST), Jorhat 785006, Assam, India

[3]Coal and Energy group (C&E), CSIR-North East Institute of Science and Technology (CSIR-NEIST), Jorhat 785006, Assam, India

[4]AcSIR-Academy of Scientific and Innovative Research, Ghaziabad, Uttar Pradesh 201002

*Electronic address: rituraj.konwar@neist.res.in.

List of all authors' email addresses: KR (kallolroy96@gmail.com); PK (parthivkar@gmail.com); SH (saikat.haldar@neist.res.in); SD (dinamani6sarangthem@gmail.com); SN (nagamaniselvaraman@gmail.com); HKB (drhkb008@gmail.com); BKS (bksaikia@neist.res.in); RK (rituraj.konwar@neist.res.in)

Corresponding author:

Rituraj Konwar, B.V.Sc., M.V.Sc., Ph.D.

Senior Principal Scientist, Centre for Preclinical Studies,

CSIR-North East Institute of Science and Technology (NEIST),

Jorhat, Assam 785006, India

Professor, AcSIR-Academy of Scientific and Innovative Research,

Ghaziabad, Uttar Pradesh 201002

Email: rituraj.konwar@neist.res.in

Mobile: +91-9451408487

Supporting Information Contents

- **Number of pages:** 10
- **Figures:** Supplemental Figures S1-S4
- **Table:** Supplemental Table S1

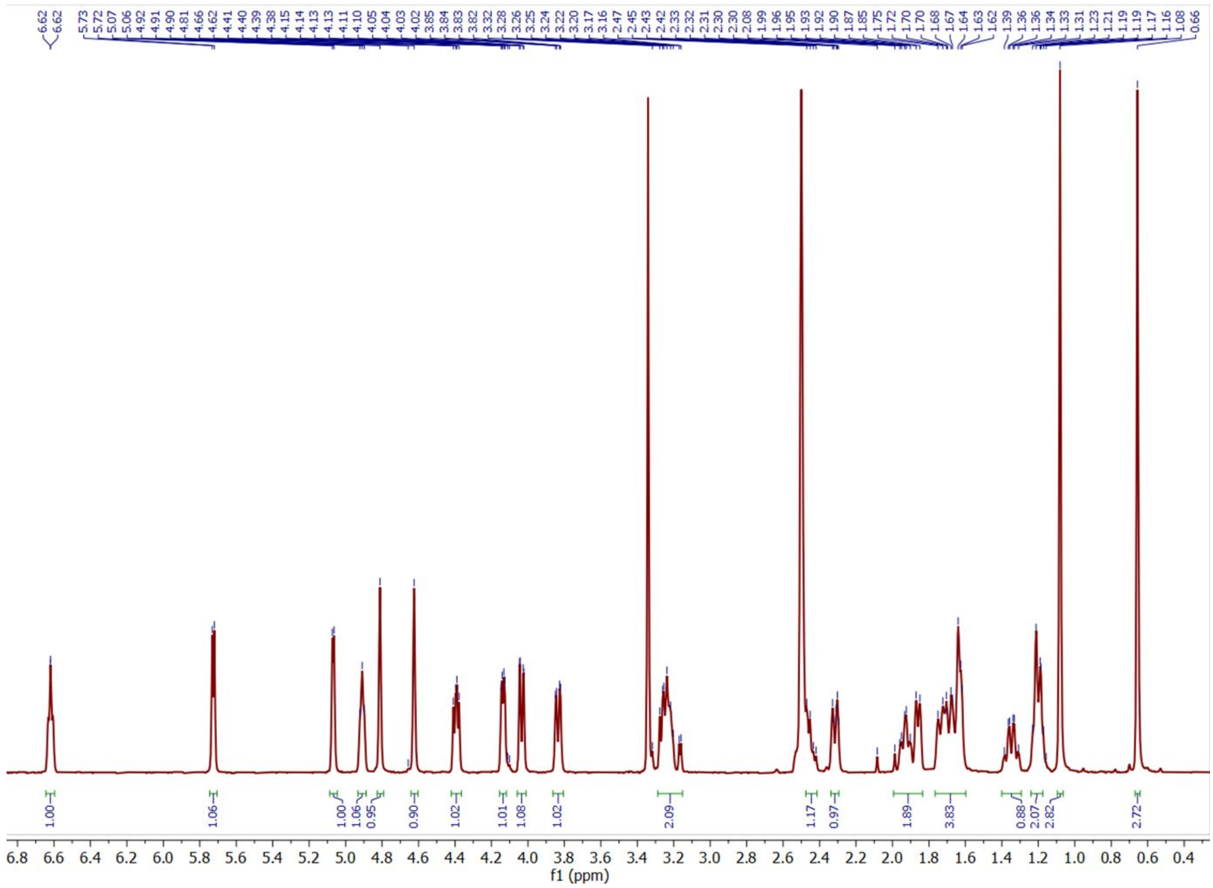

Figure S1. ^{1}H-NMR spectra of isolated AGL

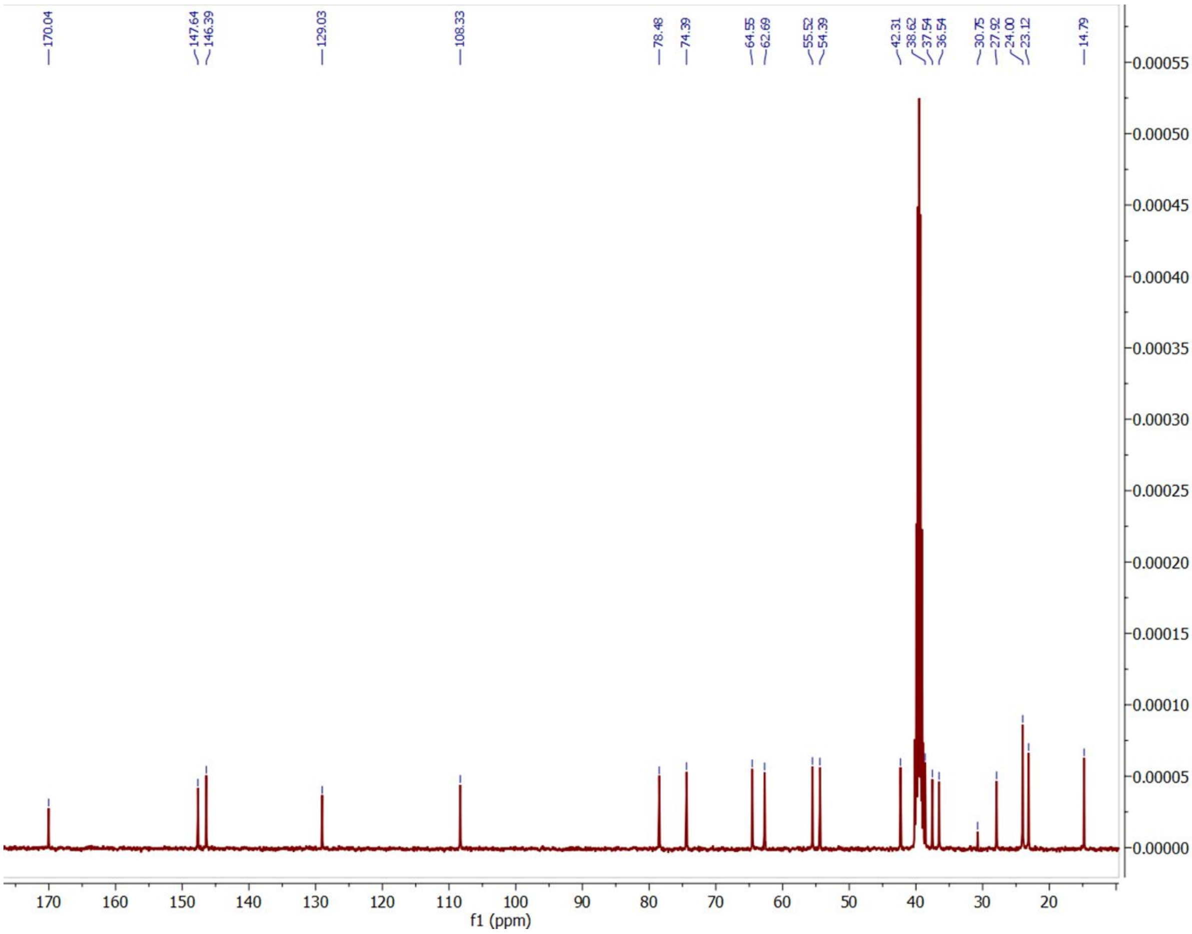

Figure S2. ^{13}C NMR spectra of isolated AGL

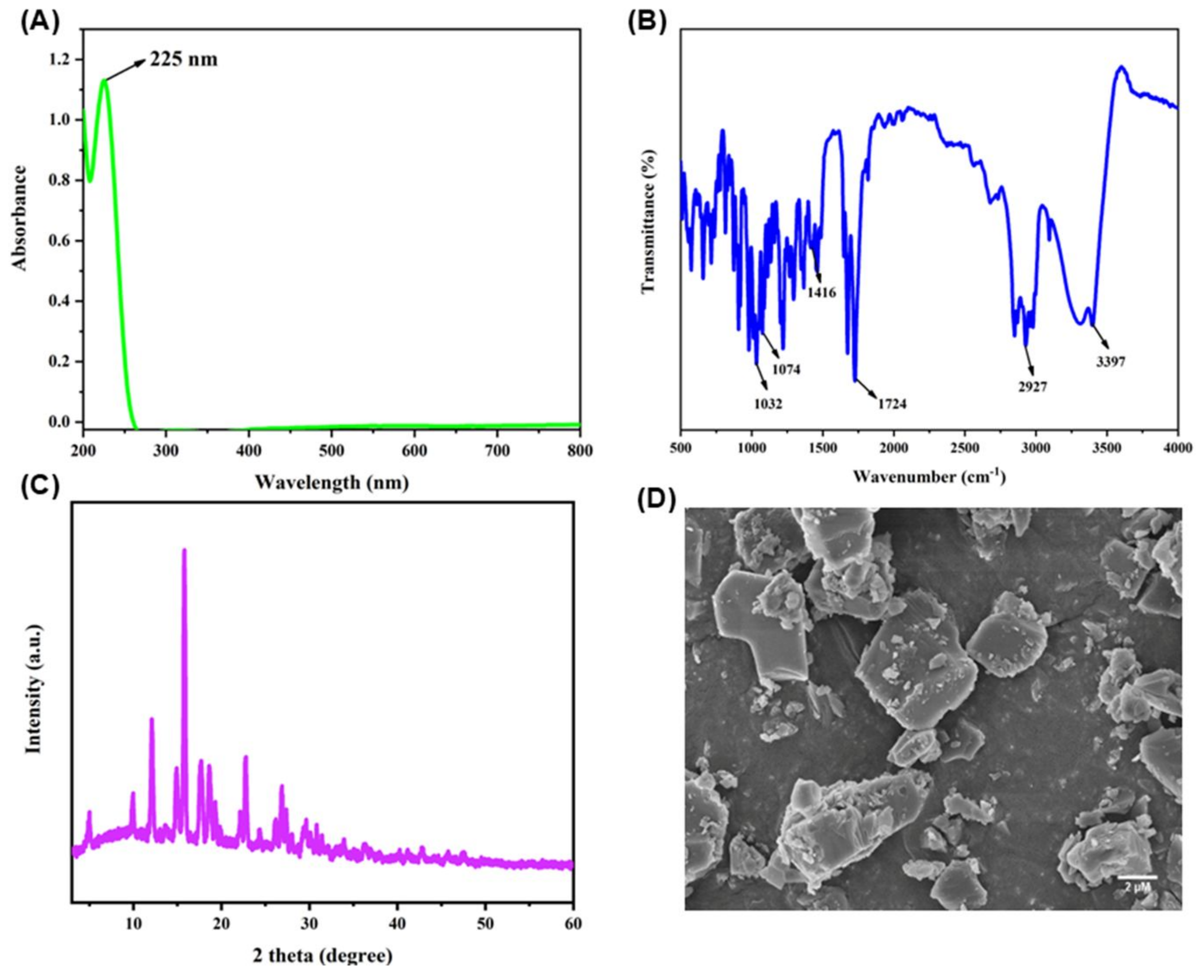

Figure S3. Physico-chemical characterizations of isolated AGL. (A)UV-VIS absorption spectra of AGL showed sharp peak at 225 nm. (B)Various functional groups and type of bonds of AGL was analysed by FTIR. AGL exhibited stretching vibrations (cm^{-1}) at 3397 (O-H), 2927 (CH_2), 1724 (lactone group), 1416 (O-H)1032 and 1074 (C-O).(C)Crystalline nature of AGL analyzed by XRD showed different peaks at 2θ = 9.94°, 12.12°, 14.94°, 15.82°, 17.72°, 18.62°, and 22.82°. (D) SEM images displayed heterogenous structure of AGLwith smooth regular appearance.

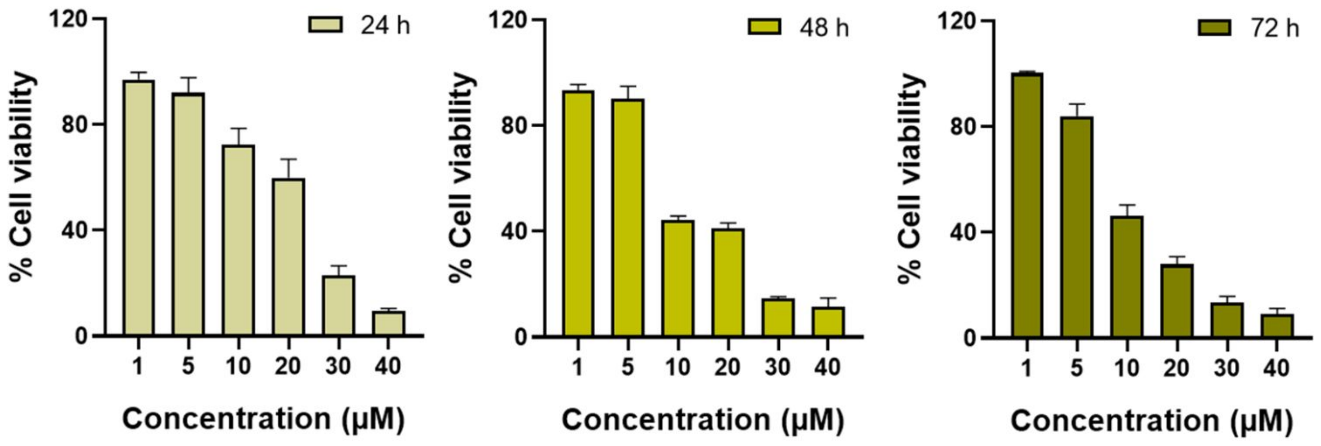

Figure S4. Effects of AGL on LA7 BC cells cytotoxicity analyzed by MTT assay. Cells were treated with various concentrations (1-40 μM) of AGL for 24 h, 48 h, and 72 h. The data are presented as mean ± SD (n = 9).

Table S1. List of identified LA7 specific genes expression datasets from literatures mining.

Expressed proteins	References
MDM2	(1)
ERβ	(2)
PCNA	(3), (7), (10), (12)
KI67	(3), (12)
BCL-2	(3), (7), (9), (12)
CD29	(4), (12)
CD49	(4), (13)
P-FOXO3a	(4)
CD133	(4), (13)
CD44	(4), (13), (16)
P21/WAF1	(4), (13)
NANOG	(5), (11)
OCT4	(5), (11)
SOX2	(5), (11)
133 (EMP3)	(6)
CCND1	(7)
C-MYC	(7), (11)
HER2	(7)
RAS	(7)
ERK1/2	(7)
AKT	(7), (20)
Ral A	(7)
STAT3	(7)
NF-κB	(7)
PKCα	(8)
Erα	(8)
GCH1	(10)
Spr	(10)
CD49f	(13)
VEGF	(14)
CX43	(15)
TGF	(17)
GATA3	(18)
GLI2/3	(18)
MDK	(19)
SREBF1	(19)
TBX3	(20)
IFITM3	(21)
β-catenin	(21)

References

(1) Bakar, N. A. A.; Ibrahim, T. A. T.; Shalan, N. A. M.; Mohamed, S. Changes in rats' breast tumor ultrastructure and immune and messenger RNA responses caused by dietary Seaweed (Kappaphycus alvarezii) extract. *J. Microsc. Ultrastruct.* **2017,** *5* (2), 70-81.

(2) Oyenihi, O. R.; Krygsman, A.; Verhoog, N.; De Beer, D.; Saayman, M. J.; Mouton, T. M.; Louw, A. Chemoprevention of LA7-induced mammary tumor growth by SM6Met, a well-characterized cyclopia extract. *Front. Pharmacol.* **2018,** *9*, 650.

(3) Karimian, H.; Fadaeinasab, M.; Zorofchian Moghadamtousi, S.; Hajrezaei, M.; Razavi, M.; Safi, S. Z.; Ameen Abdulla, M.; Mohd Ali, H.; Ibrahim Noordin, M. Chemopreventive activity of Ferulago angulate against breast tumor in rats and the apoptotic effect of polycerasoidin in MCF7 Cells: A bioassay-guided approach. *PLoS One* **2015,** *10* (5), e0127434.

(4) Zucchi, I.; Astigiano, S.; Bertalot, G.; Sanzone, S.; Cocola, C.; Pelucchi, P.; Bertoli, G.; Stehling, M.; Barbieri, O.; Albertini, A.; Schöler, H. R. Distinct populations of tumor-initiating cells derived from a tumor generated by rat mammary cancer stem cells. *PNAS* **2008,** *105* (44), 16940-16945.

(5) Pourbagher, R.; Ghorbani, H.; Akhavan-Niaki, H.; Jorsaraei, S. G. A.; Fattahi, S.; Ghooran, S.; Abedian, Z.; Ghasemi, M.; Saeedi, F.; Jafari, N.; Kalali, B. Downregulation of stemness genes and induction of necrosis in rat LA7 cancer stem cells induced tumors treated with starved fibroblasts culture supernatant. *Rep. Biochem. Mol. Biol.* **2021,** *10* (1), 105.

(6) Zucchi, I.; Montagna, C.; Susani, L.; Montesano, R.; Affer, M.; Zanotti, S.; Redolfi, E.; Vezzoni, P.; Dulbecco, R. Genetic dissection of dome formation in a mammary cell line: identification of two genes with opposing action. *PNAS* **1999,** *96* (24), 13766-13770.

(7) Vela, E.; Escrich, E. Molecular profiling and malignant behavior define two rat mammary tumor cell lines as a relevant experimental model. *J. Cell. Biochem.* **2016,** *117* (12), 2825-2834.

(8) Arun, A.; Ansari, M. I.; Popli, P.; Jaiswal, S.; Mishra, A. K.; Dwivedi, A.; Hajela, K.; Konwar, R. New piperidine derivative DTPEP acts as dual-acting anti-breast cancer agent by targeting ER α and downregulating PI 3K/Akt-PKC α leading to caspase-dependent apoptosis. *Cell Prolif.* **2018,** *51* (6), e12501.

(9) Saini, K. S.; Hamidullah; Ashraf, R.; Mandalapu, D.; Das, S.; Siddiqui, M. Q.; Dwivedi, S.; Sarkar, J.; Sharma, V. L.; Konwar, R. New orally active DNA minor groove binding small molecule CT-1 acts against breast cancer by targeting tumor DNA damage leading to p53-dependent apoptosis. *Mol. Carcinog.* **2017,** *56* (4), 1266-1280.

(10) Ibrahim, M. Y.; Hashim, N. M.; Omer, F. A. A.; Abubakar, M. S.; Mohammed, H. A.; Salama, S. M.; Jayash, S. N. Potential antitumor effect of α-mangostin against rat mammary gland tumors induced by LA7 cells. *Int. J. Mol. Sci.* **2023,** *24* (12), 10283.

(11) Pourbagher, R.; Akhavan-Niaki, H.; Jorsaraei, S. G. A.; Fattahi, S.; Sabour, D.; Zabihi, E.; Abedian, Z.; Ghasemi, M.; Golpour, M.; Mostafazadeh, A. Targeting LA7 breast cancer stem cells of rat through repressing the genes of stemness-related transcription factors using three different biological fluids. *Gene* 2020. ***734*,** 144381.

(12) Karimian, H.; Fadaeinasab, M.; Moghadamtousi, S. Z.; Hajrezaei, M.; Zahedifard, M.; Razavi, M.; Safi, S. Z.; Mohan, S.; Khalifa, S. A.; El-Seedi, H. R.; Abdulla, M. A. The chemopreventive effect of Tanacetum polycephalum against LA7-induced breast cancer in rats and the apoptotic effect of a cytotoxic sesquiterpene lactone in MCF7 cells: a bioassay-guided approach. *Cell. Physiol. Biochem.* **2015,** *36* (3), 988-1003.

(13) Zucchi, I.; Sanzone, S.; Astigiano, S.; Pelucchi, P.; Scotti, M.; Valsecchi, V.; Barbieri, O.; Bertoli, G.; Albertini, A.; Reinbold, R. A.; Dulbecco, R. The properties of a mammary gland cancer stem cell. *PNAS* **2007,** *104* (25), 10476-10481.

(14) Fan, X.; Krzyzanski, W.; Wong, R. S.; Liu, D.; Yan, X. Novel combination of erythropoietin and romiplostim to treat chemotherapy-induced anemia and thrombocytopenia via pharmacodynamic interaction on hematopoietic stem and progenitor cells. *ACS Pharmacol. Transl. Sci.* **2023,** *6* (12), 1884-1897.

(15) Ehmann, U. K.; Calderwood, S. K.; Stevenson, M. A. Gap-junctional communication between feeder cells and recipient normal epithelial cells correlates with growth stimulation. *In Vitro Cell. Dev. Biol. Animal* **2001,** *37* (2), 100-110.

(16) Sharma, M.; Mittapelly, N.; Banala, V. T.; Urandur, S.; Gautam, S.; Marwaha, D.; Rai, N.; Singh, N.; Gupta, A.; Mitra, K.; Mishra, P. R. Amalgamated microneedle array bearing ribociclib-loaded transfersomes eradicates breast cancer via CD44 targeting. *Biomacromolecules* **2022,** *23* (3), 661-675.

(17) Ehmann, U. K., DeVries, J. T.; Chen, M. S. C.; Adamos, A. A.; Guzman, R. C.; Omary, M. B. An in vitro model of epithelial cell growth stimulation in the rodent mammary gland. *Cell Prolif.* **2003,** *36* (4), 177-190.

(18) Mosca, E.; Bertoli, G.; Piscitelli, E.; Vilardo, L.; Reinbold, R. A.; Zucchi, I.; Milanesi, L. Identification of functionally related genes using data mining and data integration: a breast cancer case study. *Bmc Bioinformatics* **2009,** *10* (Suppl 12), S8.

(19) Yan, P.; Jimenez, E. R.; Li, Z.; Bui, T.; Seehawer, M.; Nishida, J.; Foidart, P.; Stevens, L. E.; Xie, Y.; Gomez, M. M.; Park, S. Y. Midkine as a driver of age-related changes and increase in mammary tumorigenesis. *Cancer Cell* **2024,** *42* (11), 1936-1954.

(20) Platonova, N. Scotti, M.; Babich, P.; Bertoli, G.; Mento, E.; Meneghini, V.; Egeo, A.; Zucchi, I.; Merlo, G. R. TBX3, the gene mutated in ulnar-mammary syndrome, promotes growth of mammary epithelial cells via repression of p19ARF, independently of p53. *Cell Tissue Res.* **2007,** *328* (2), 301-316.

(21) Arbab, I. A. Zerumbone (ZER), a Potential Anticancer for Breast Mediates Cancer Cell Death Through Targeting β-catenin Signaling Pathway in Tumor Regression in Sprague Dawley Rat Mammary Gland Tumors. *Sch Int. J. Tradit. Complement. Med*. **2023,** *6* (10), 149-160.
